# Genetic insights into foveal morphology and its associations with pigmentation and age-related macular degeneration

**DOI:** 10.1101/2025.06.27.25330434

**Published:** 2025-06-28

**Authors:** David J. Green, David Romero-Bascones, Thomas H. Julian, Sofia Torchia, Heer N.V. Joisher, UK Biobank Eye and Vision Consortium, Unai Ayala, Maitane Barrenechea, Jay E. Self, Graeme C. Black, Tomas Fitzgerald, Ewan Birney, Constance L. Cepko, Joseph Carroll, Panagiotis I. Sergouniotis

**Affiliations:** 1 Division of Evolution, Infection and Genomics, School of Biological Sciences, Faculty of Biology, Medicine and Health, University of Manchester, Manchester, UK.; 2 Biomedical Engineering Department, Faculty of Engineering (MU-ENG), Mondragon Unibertsitatea, Mondragón, Spain.; 3 Manchester Royal Eye Hospital, Manchester University NHS Foundation Trust, Manchester, UK.; 4 Christabel Pankhurst Institute, The University of Manchester, Manchester, UK; 5 European Molecular Biology Laboratory, European Bioinformatics Institute (EMBL-EBI), Wellcome Genome Campus, Cambridge, UK; 6 Department of Genetics, Blavatnik Institute, Boston, MA, USA; 7 Department of Ophthalmology, Harvard Medical School, Boston, MA, USA; 8 Howard Hughes Medical Institute, Chevy Chase, MD, USA; 9 Clinical and Experimental Sciences, Faculty of Medicine, University of Southampton, Southampton, UK; 10 Southampton Eye Unit, University Hospital Southampton NHS Foundation Trust, Southampton, UK; 11 Manchester Centre for Genomic Medicine, Saint Mary’s Hospital, Manchester University NHS Foundation Trust, Manchester, UK; 12 Cell Biology, Neurobiology and Anatomy, Medical College of Wisconsin, Milwaukee, WI, USA; 13 Ophthalmology and Visual Sciences, Medical College of Wisconsin, Milwaukee, WI, USA

## Abstract

The fovea is the small depression at the neurosensory retina that underlies high-resolution central vision. It is vulnerable to disease and disruption of its architecture causes visual disability. Foveal morphology varies significantly across individuals. The molecular causes and functional consequences of this anatomical diversity are incompletely understood. Here, we extracted six foveal morphological parameters from Optical Coherence Tomography (OCT) images of 55,604 UK Biobank participants. We found notable variability in foveal morphology, and detected significant links with sex and genetic ancestry. Genome-wide association studies identified 197 lead loci across the six foveal morphological parameters, implicating genes involved in pigmentation (e.g., *TYR*, *TSPAN10, GPR143*) and patterning (e.g., *FGFR2*, *PTPRD, CYP1A1*). Heritability estimates ranged from 29–43%. Foveal pit volume was associated with future risk of age-related macular degeneration (HR>3.5, p=0.001), a finding supported by Mendelian randomization. These results establish foveal morphology as a highly-heritable trait with substantial influence over retinal disease risk.

## INTRODUCTION

The fovea is a specialized structure at the center of the retina. It has a key role in high-acuity vision, supports most daily life activities, and enables tasks such as reading and face recognition.^1,2^ The fovea is affected in a range of retinal disorders, including common adult-onset diseases such as age-related macular degeneration (AMD) (which is associated with loss of foveal cells) and pediatric conditions like albinism (which is characterized by foveal hypoplasia).^2^

The fovea has been the subject of study for more than a century. Despite this, there are significant gaps in our knowledge, partly because animal models that are routinely used in research (e.g. most murine and teleost models) lack this morphological specialization.^2^ Recent advances in genomics, data science and *in vivo* imaging technologies have transformed our ability to study complex biological structures like the fovea.^3^ Another major enabler has been the increasing availability of large-scale cohorts with a rich phenotypic scope. One example is the UK Biobank, a biomedical resource containing in-depth genetic and health information from >500,000 individuals.^4^ Many UK Biobank participants underwent enhanced phenotyping including visual function assessment (>130,000 volunteers) and imaging of the central retina (>84,000 volunteers);^5^ the latter was obtained using optical coherence tomography (OCT), a non-invasive imaging modality that rapidly generates cross-sectional retinal scans at micrometre-resolution.^6^

The most prominent morphological feature of the human fovea is the pit, corresponding to a central excavation of the inner retinal layers. Previous imaging studies have highlighted substantial morphological variability in respect to the depth, width and volume of the pit.^1,7–11^ This anatomical diversity is thought to arise from variations in foveal development. Indeed, a blunted or absent foveal pit is often seen in individuals born prematurely.^12^ Differences in foveal morphological parameters (hereafter, foveal traits) have also been reported between male and female and among individuals with different ancestral backgrounds.^7,13,14^

**Fig. 1.**
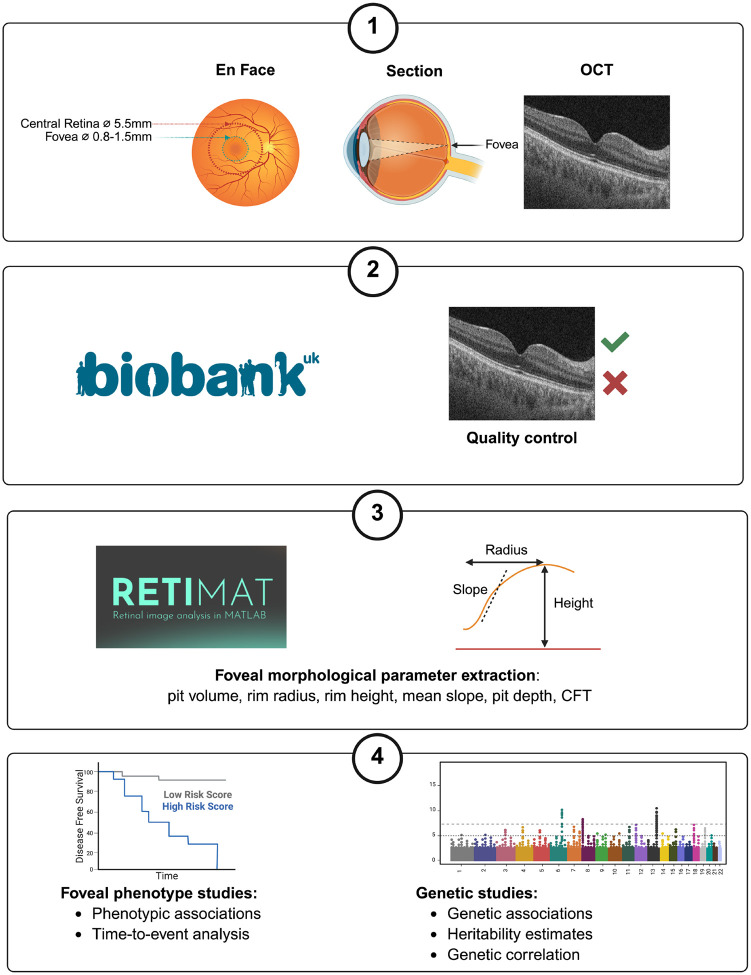
Outline of the study design. First, we used the RETIMAT tool to extract six key foveal traits from UK Biobank OCT scans (pit volume, rim radius, rim height, mean slope, pit depth and CFT). Subsequently, we performed phenotypic and genetic studies to gain insights into these foveal traits. Phenotypic analyses included the investigation of the relationships between the studied foveal traits and age, spherical equivalent refractive error, sex, genetic ancestry, and visual acuity. Time-to-event analyses involved assessing the relationships between the six studied foveal traits and two major causes of visual impairment, age-related macular degeneration and glaucoma. Genetic investigations included common-variant genome-wide association studies (primary and replication), heritability estimation, and genetic correlation analyses. OCT, optical coherence tomography; CFT, central foveal thickness.

Previous studies have sought to investigate the genetic architecture of OCT-derived retinal traits, including central retinal thickness, and inner and outer retinal layer thickness.^15–20^ However, to date, a comprehensive analysis of the genetic determinants of foveal morphology has not been undertaken. Here, we sought to address this knowledge gap and to explore the association between foveal traits and common retinal conditions (Fig.1).

## RESULTS

### Phenotypic variability in foveal traits

After applying standard OCT quality control filters,^20^ we defined a subset of the UK Biobank population with high-quality OCT scans. This cohort included 55,604 individuals and had similar sex and age profile to the overall UK Biobank population.^15^ Most study subjects were female (52%) and the mean age at OCT imaging was 57.7 years (standard deviation: 8.1 years).

**Fig. 2.**
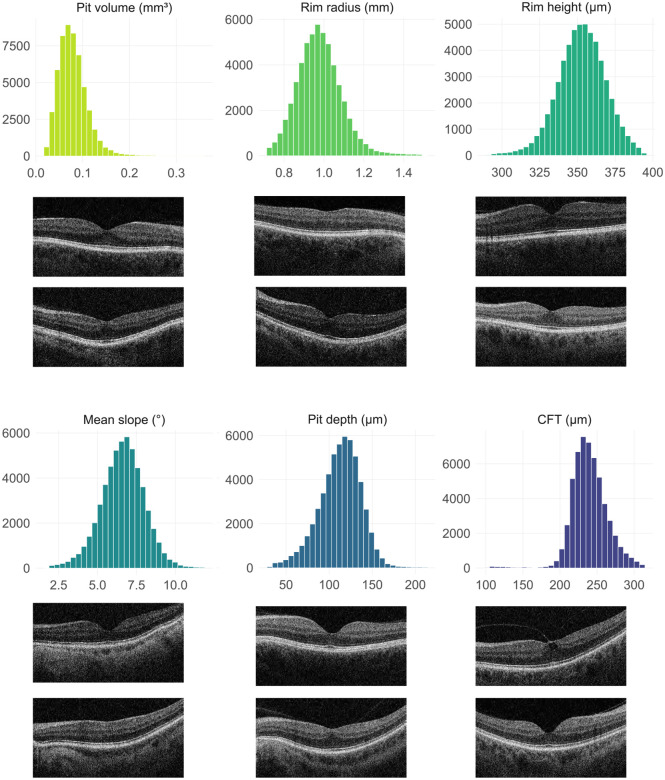
Histograms showing the distribution of six foveal traits in UK Biobank participants (after removal of 1% from the upper and lower tails of the data). Also shown are six examples of OCT scans from the extreme ends of each distribution. The following mean values were obtained: pit volume 0.080 mm^3^ (range: 0.024–0.356 mm^3^), rim radius 0.98 mm (range: 0.71–1.49), rim height 353 μm (range: 291–394 μm), mean slope 6.61° (range: 1.88–11.86°), pit depth 113 μm (range: 32–213 μm), central foveal thickness (CFT) 240 μm (range: 102–312 μm).

For each study subject, an OCT ‘volume scan’ (containing 128 cross-sectional images) was available from the central retina of each eye. We used left eye images as input to RETIMAT, an automated pipeline designed to extract foveal traits from OCTs.^14^ We focused on the following six parameters: foveal pit volume, rim radius, rim height, mean slope, pit depth and central foveal thickness (CFT) (Fig.1). The greatest fold range across UK Biobank participants was observed for pit volume (15.1-fold), followed by pit depth (6.5-fold) and mean slope (6.3-fold), indicating large differences between the most extreme phenotypes in the population. In contrast, parameters such as rim height (1.4-fold) and rim radius (2.1-fold) were more constrained (Fig.2).

**Fig. 3.**
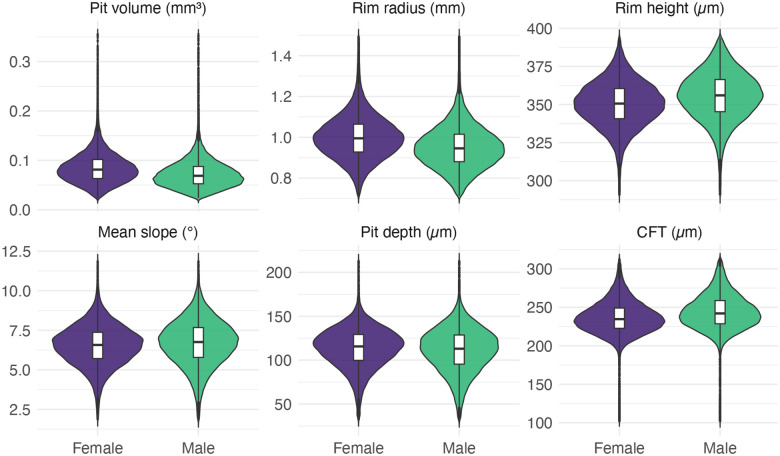
Violin plots showing the distribution of six foveal traits between male and female UK Biobank participants. Females tend to have larger pit volumes (0.085 mm^3^ vs. 0.073 mm^3^), deeper pits (114 μm vs. 111 μm) and slightly larger rim radii (1.00 vs. 0.954) than male. In contrast, male had higher central foveal thickness (CFT) (244 μm vs. 236 μm), steeper mean pit slopes (6.69° vs. 6.54°) and greater rim height (355 μm vs. 350 μm) than females. The Wilcoxon signed rank test was used to make comparisons and all p-values were found to be < 2.3 × 10^−10^. It is noted that the units for the foveal traits differ and, as such, the scales should not be directly compared. Further information, including numerical data, can be found in Supplementary Table S3.

**Fig. 4.**
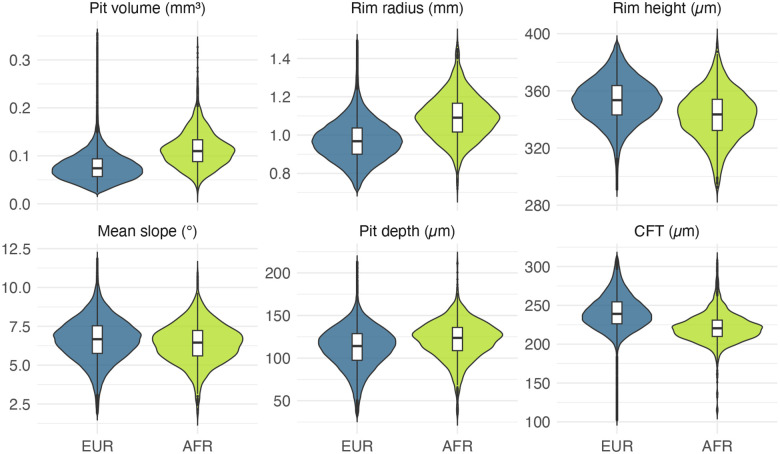
Violin plots showing the distributions of six foveal traits in individuals with European- and African-like genetic ancestries (EUR and AFR respectively). The AFR group had notably larger pit volumes (0.11 mm^3^ vs. 0.08 mm^3^), deeper foveal pits (121.37 μm vs. 112.19 μm) and greater rim radii (1.09 mm vs. 0.97 mm) than the EUR group. In contrast, the EUR group had higher central foveal thickness (240.65 μm vs 221.26 μm), steeper mean pit slope (6.62 ° vs. 6.41 °) and greater rim height (353.17 μm vs. 342.80 μm) than the AFR group. The Wilcoxon signed rank test was used to make comparisons and all p-values were found to be < 1 × 10^−10^. It is noted that he units for the foveal traits differ and, as such, the scales should not be directly compared. Further information, including numerical data, can be found in Supplementary Table S4.

First, we explored associations between the six studied foveal traits and age at image acquisition and found no notable correlations (Supplementary Table S1). We then examined associations between the same six foveal traits and spherical equivalent refractive error (a proxy for eye size) and found these to be variably correlated (Supplementary Table S2). Next, we inspected sex-related differences in foveal morphology, identifying statistically significant variation for all six studied traits. On average, females were found to have deeper, broader and larger foveal pits than males (Fig.3; Supplementary Table S3). We also compared UK Biobank participants of different genetic ancestries, focusing primarily on individuals of European- and African-like ancestries (as these are the two largest groups in the dataset and the only two to contain >1,000 individuals; Fig.4; Supplementary Fig.S1; Supplementary Table S4). Again, we found statistically significant differences for all six studied foveal traits. On average, individuals of African-like genetic ancestries had deeper, broader and larger foveal pits that their European counterparts. These findings reinforce the role of sex and ancestry in shaping foveal morphology, and highlight the importance of including these parameters as covariates in subsequent genetic and phenotypic analyses of foveal traits.

**Fig. 5.**
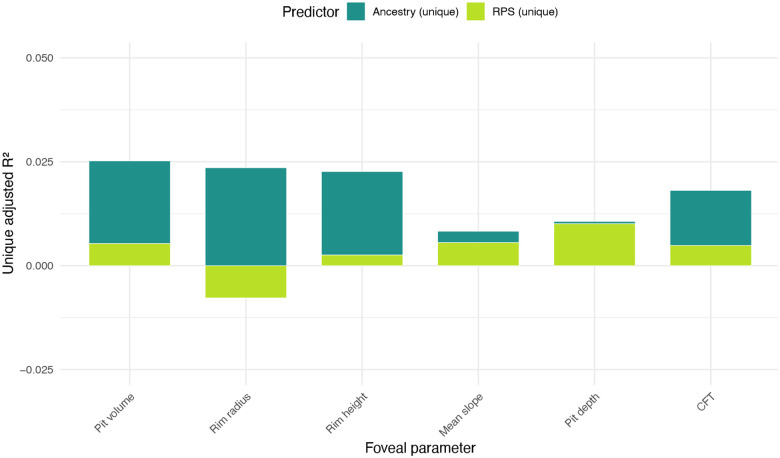
Unique contributions of fundus pigmentation and genetic ancestry to foveal morphology. Bar plots show the unique adjusted R2 values for the six studied foveal traits, representing the proportion of variance explained by Retinal Pigment Score (RPS) and genetic ancestry, after adjusting for age, sex and spherical equivalent refractive error. Unique R2 values were calculated from linear models by subtracting the R2 of ancestry-only or RPS-only models from the combined model. Genetic ancestry accounted for a larger share of variance across most traits, particularly rim radius and pit volume (2.7% and 2.4% of variance, respectively). Pigmentation (RPS) explained modest additional variance, most notably for rim height and pit depth (0.9% and 0.6% of variance, respectively). For some traits, such as rim radius, the unique contribution of RPS was slightly negative, likely reflecting noise or model penalization rather than a true inverse effect. Further information, including numerical data, can be found in Supplementary Table S5.

Aiming to gain insights into the factors underlying the detected differences between ancestral groups, we investigated the role of fundus (principally choroidal) pigmentation. To evaluate this, we used a fundus imaging-derived proxy of pigmentation, the ‘Retinal Pigment Score’ (RPS).^21^ We then calculated the unique variance explained (adjusted R2) values by RPS and ancestry across the six studied foveal traits, adjusting for age, sex and spherical equivalent refractive error. Overall, genetic ancestry explained a greater proportion of trait variance than fundus pigmentation for most traits, particularly pit volume and rim radius. It is noted that pigmentation explained negligible unique variance for rim radius, suggesting little independent contribution beyond genetic ancestry (Fig.5; Supplementary Table S5).

To assess the extent to which foveal morphology predicts visual acuity, we performed linear regression with LogMAR vision as the outcome, and age, sex, spherical equivalent refractive error, and genetic ancestry as covariates. Several traits were significantly associated with acuity, but all effect sizes were small (R2 < 1.3%). Mean slope was the strongest predictor, with steeper pits linked to better vision. In contrast, greater pit volume was associated with worse acuity, and central foveal thickness showed no effect (p = 0.30) (Supplementary Fig.S2).

### Association between foveal traits and risk of retinal disease

To investigate whether foveal morphology constitutes a risk factor for future retinal conditions, we performed Cox proportional hazards modeling using the six studied foveal traits, along with time-to-event data for two leading causes of visual impairment, AMD and glaucoma. We adjusted for age, sex, spherical equivalent refractive error, and genetic ancestry in these analyses.

**Fig. 6.**
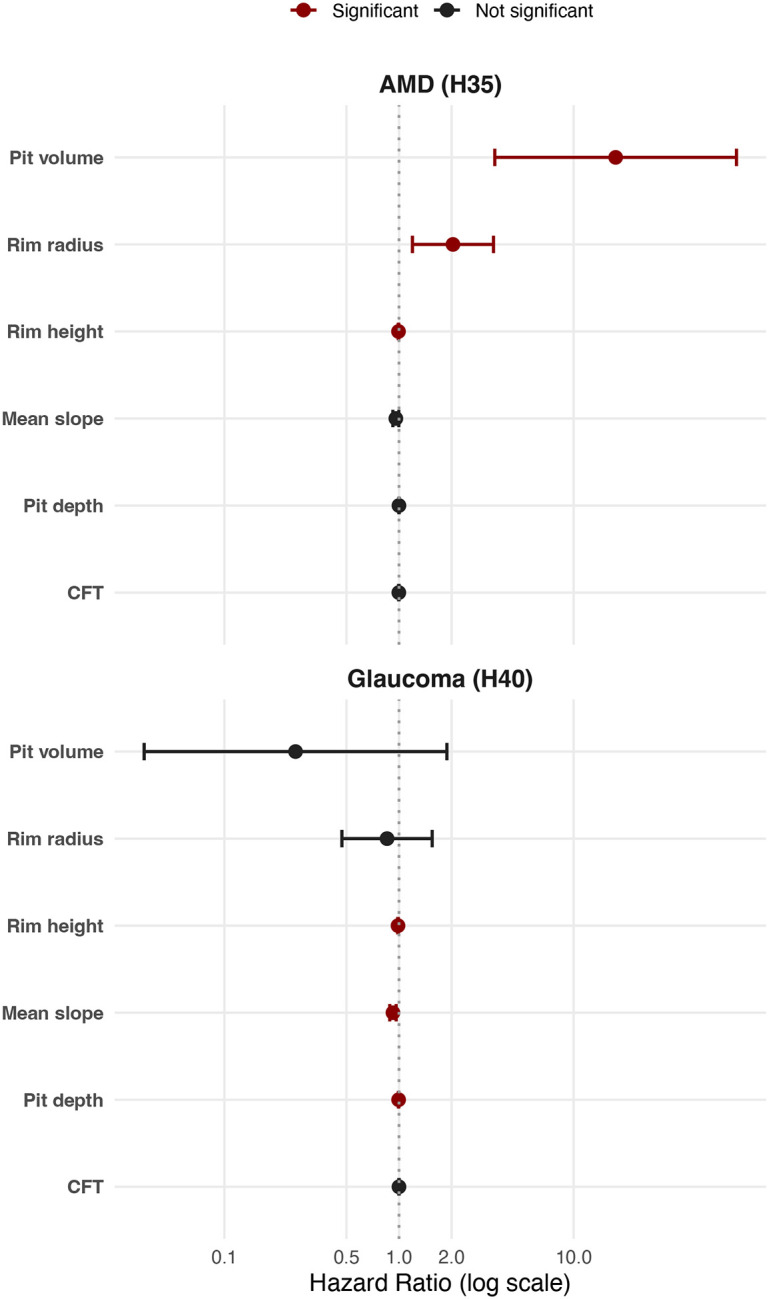
Cox regression analysis of associations between foveal traits and post-OCT-scan assigned codes for age-related macular degeneration (AMD) (H35) and glaucoma (H40). Statistically significant associations (after correcting for multiple comparisons using a 5% false-discovery rate [FDR] threshold) are highlighted with red. Further information, including numerical data, can be found in Supplementary Table S6.

For AMD (n=1,345 cases), there was a notable association with larger pit volume (hazard ratio 17.4; 95% confidence intervals [CI] 3.5–85.7) and wider rim radius (hazard ratio 2.0; 95% CI 1.2–3.5). For glaucoma (n=1,166 cases), there were statistically significant associations with mean slope, rim height and pit depth. However, the corresponding hazard ratios indicated only small effect sizes (ranging from 0.255 to 0.999) (Fig.6, Supplementary Table S6).

### Genetic associations of foveal traits

To investigate the genetic factors influencing the six studied foveal traits, we performed common-variant genome-wide association studies (GWAS) using REGENIE.^22^ For these analyses, we defined a subset of the UK Biobank population that can be considered genetically well-mixed (*i.e*. includes participants that were assigned by genotype principal component analysis to a cluster with subjects of mostly European-like ancestries). After applying relevant imaging, genetic, and foveal trait quality controls, this cohort included 36,205 individuals; 29,710 of these were recruited between 2006 and 2010 (Instance 0, ‘Initial assessment’) and were used in the primary analysis while 6,495 were independently recruited between 2012 and 2013 (Instance 1, ‘First repeat assessment visit’) and were included in the replication study. The following covariates were incorporated into the models: age, sex, height, weight, spherical equivalent refractive error, and genetic principal components 1 to 20. We utilized a conventional threshold for multiple testing correction in GWAS studies, p < 5 × 10^−8^ (‘genome-wide significance’).

**Fig. 7.**
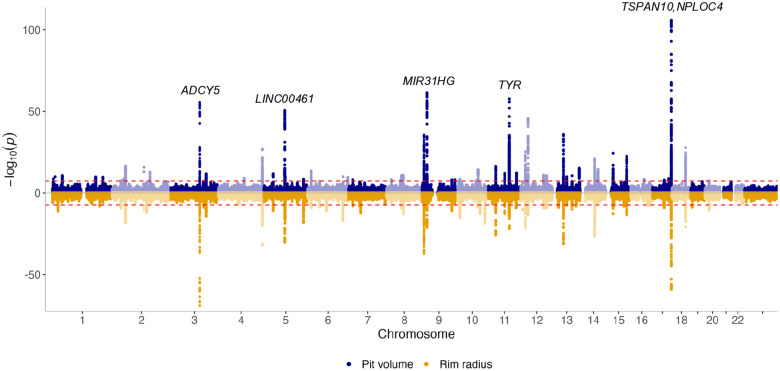
A Miami plot, illustrating the results of the primary genome-wide association study for foveal pit volume (top) and rim radius (bottom). The red line indicates the threshold for genome-wide significance, and 5 key loci exceeding this value have been annotated.

Genome-wide significant variants in the primary GWAS included: 5,933 for pit volume; 6,251 for rim radius; 7,694 for rim height; 4,117 for mean slope; 4,710 for pit depth; and 2,723 for CFT. Following analysis with GCTA-COJO (conditional and joint multiple-variant analysis),^23^ these merged into the following lead loci: 64 for pit volume, 65 for rim radius, 45 for rim height, 29 for mean slope, 40 for pit depth, and 28 for CFT (Fig.7, Supplementary Fig.S3–8; Supplementary Dataset S1). We subsequently performed a replication GWAS using UK Biobank subjects that were not included in the primary analysis, establishing a high level of concordance between the primary and replication studies with all traits having a correlation coefficient greater than 0.9 (Supplementary Fig.S9–14). Although the replication sample was considerably smaller, several lead loci still reached genome-wide significance in both studies: 6 for pit volume, 4 for rim radius, 1 for rim height, 1 for mean slope, 2 for pit depth, and 2 for CFT (Supplementary Dataset S1).

We identified a combination of trait-specific and shared loci influencing foveal morphology. Many of these loci encompass variants previously linked to retinal layer thickness parameters (including *TSPAN10* and *LINC00461*) while a subset of them has been linked to monogenic disorders, most notably, albinism (including *TYR*, *OCA2* and *GPR143*) (Table 1 and Supplementary Dataset S1). Our primary analysis highlighted several loci that have not been previously associated with retinal phenotypes, including *CYP1A1, PPP1R9A, KMT2A, CASZ1* and *CCDC122*. We also identified variants in *FGFR2*, *PTPRD*, *SPRY2, FLT1 and LATS1*, for which associations have only been detected for retinal thickness measurements but not for foveal/macular traits.

MAGMA gene set enrichment analysis did not yield any statistically significant hits after correction for multiple testing.

It is noted that the genomic inflation factors (λGC) ranged from 1.12 to 1.17, with corresponding linkage disequilibrium (LD) score intercepts close to 1 (range: 1.005–1.027). These results suggest minimal confounding from population structure or cryptic relatedness. The attenuation ratios were uniformly low (all < 0.11), consistent with polygenic architecture and well-controlled inflation across all six studied traits (Supplementary Table S7).

### Heritability estimates for foveal traits

We used linkage disequilibrium (LD) score regression analysis^24^ to obtain heritability estimates for the six studied foveal traits. We found moderate to high heritability, ranging from h2 = 0.29 (standard error [SE] = 0.03) for CFT to h2 = 0.43 (SE = 0.04) for pit volume. Rim height (h2 = 0.41), rim radius (h2 = 0.39), pit depth (h2 = 0.34), and mean slope (h2 = 0.31) were also significantly heritable (Supplementary Table S7).

### Global genetic correlation between foveal traits

LD score regression was also used to assess the global genetic correlation between the studied traits. We found significant interdependence between many foveal traits. The strongest correlations (rg > 0.8) were observed between pit depth and mean slope (rg = 0.88) and between pit volume and rim radius (rg = 0.85), suggesting that these pairs reflect closely related biological processes. Moderate correlations (rg = 0.6–0.8) included pit depth and pit volume (rg = 0.76), mean slope and rim height (rg = 0.65), and pit depth and rim height (rg = 0.57), indicating shared underlying variation across several morphological aspects of the fovea.

**Fig. 8.**
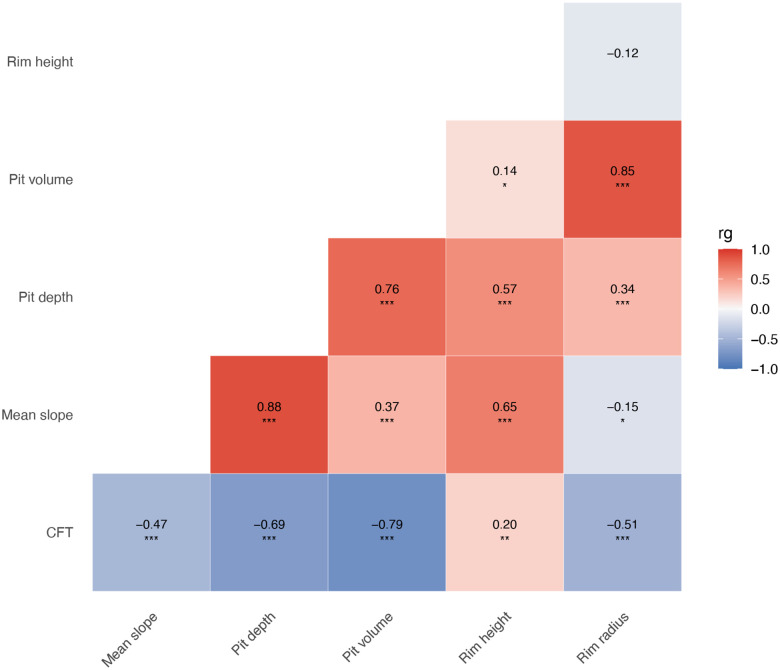
Genetic correlations between foveal traits. Asterisks indicate levels of statistical significance (* p < 0.05, ** p < 0.01, *** p < 0.001). Further information, including numerical data, can be found in Supplementary Table S8. CFT, central foveal thickness.

In contrast, correlations between CFT and other parameters were more variable. We observed strong negative correlations between CFT and pit volume (rg = −0.79), pit depth (rg = −0.69), and rim radius (rg = −0.51), consistent with an inverse relationship between retinal thickness and pronounced foveal pit excavation (Fig.8).

Correlations between foveal traits and fundus pigmentation (RPS) were generally weak (rg = −0.23 with pit depth, −0.22 with mean slope), with the strongest positive correlation seen with CFT (rg = 0.23) (Supplementary Table S8).

### Mendelian randomization

We performed Mendelian randomization (MR) analysis on the two foveal traits that we found to be associated with significantly increased risk of AMD and glaucoma. This analysis revealed causal relationships between foveal morphology and retinal disease risk. There was evidence of causal association between increased pit volume and AMD (OR = 1.12, 95% CI: 1.04–1.21, inverse variance weighted [IVW] p = 0.004). This was robust to a leave-one-out test using the IVW method. In contrast, rim radius showed no evidence of a causal effect on AMD or glaucoma (p > 0.2 across all methods). Pit volume was also nominally associated with decreased risk of glaucoma (IVW p = 0.02; OR = 0.94, 95% CI: 0.89–0.99). This result remained significant in the MR Egger (p=0.02) and weighted mode (p=0.03) but did not quite exceed significance in the weighted median (p=0.056) nor persist throughout leave-one-out testing using the IVW method (Supplementary Table S9). Reverse Mendelian randomization analyses yielded no evidence of AMD or glaucoma influencing foveal morphology. Overall, the findings provide evidence that pit morphology causally influences the risk of AMD and primary open-angle glaucoma, but these results should be interpreted in the context of their reliance on several key assumptions of Mendelian randomization being met (see Methods for further details on this).

## DISCUSSION

In the present study, we performed large-scale phenotypic and genetic analyses to better understand the variability and determinants of foveal morphology. By leveraging retinal imaging data from UK Biobank participants and a comprehensive foveal trait calculation tool (RETIMAT), we were able to detect differences in foveal morphology influenced by sex, genetic ancestry, and genetic factors. We also found a notable association between foveal pit volume and AMD.

We first analyzed how foveal architecture varies at the population level. As the number of subjects in this study is an order of magnitude greater than in most prior reports, we were able to identify previously unrecognized variability in foveal pit morphology (e.g. 15-fold for pit volume). Sex and ancestry were found to significantly influence foveal morphology, an observation that is in keeping with the findings of previous smaller-scale studies.^7,9,13,14^ In line with previous research, males exhibited greater rim height, shorter rim radii, and a steeper mean slope.^14^ Similarly, we found that individuals with African ancestries had wider, deeper, and more voluminous foveal pits than their European counterparts, in line with findings by Zouache *et al*.^7^ The smaller East- and South-Asian ancestral groups fell between these two, consistent with the limited normative datasets available for Asian populations.^25,26^ It is highlighted that, in contrast to most previous studies, we used genetic ancestry, a metric that is more accurate and objective than ethnicity or self-reported ancestry.^27^ We also performed additional analyses to gain insights into the extent to which the observed difference between continental groups is driven by pigmentation. Our observations suggest that genetic ancestry tends to be an independent contributor irrespective of fundus pigmentation, especially for foveal pit volume and rim radius.

We found that a larger pit volume is a key risk factor for AMD, and that there is a 33% increase in risk per 0.1 mm^3^ increase in pit volume. This observation was supported by two orthogonal approaches, Cox regression analysis and Mendelian randomization. The results of the latter substantially reduce the risk that the observed association is due to reverse causation or confounding, and support the assertion that pit volume is a risk factor for AMD rather than a correlate. While the hazard ratio was 17.4 (95% CI 3.5–85.7), the corresponding odds ratio (OR) was 26.1 (95% CI 6.1–112.0, p = 1.1 × 10^−5^) (Fig.6, Supplementary Tables S6 and S10). It is noted that several environmental and genetic risk factors have been previously associated with AMD, including: age (OR 1.1), gender (OR 1.6), smoking (OR 1.9)^28^, *CFH* gene variants (e.g. homozygosity for rs1061170 corresponds to OR of 4.7)^29^, *ARMS2* locus variants (e.g. homozygosity for rs10490924 corresponds to OR of 8.6)^30^ and family history (OR 10.8).^31^ Although AMD selectively affects the foveal/macular region, to date, anatomical risk factors have been understudied^9^ and, to our knowledge, this is the first time that specific foveal morphological parameters are found to set the stage for susceptibility to AMD. It can be speculated that the link between a larger pit and AMD risk is driven by differences in the distribution of macular xanthophyll pigments and/or by alterations in photoreceptor cell densities in the vulnerable parafoveal area of low rod-to-cone ratio.^32^ Future advanced ophthalmic imaging and mathematical modelling studies will provide further insights into how foveal anatomy predisposes to this leading cause of visual impairment.

Following phenotypic analyses, including of foveal traits that have received little attention so far (such as foveal volume and radius), we performed genetic association studies. We found that the observed morphological diversity has a strong genetic underpinning, with a high level of heritability across most traits (Supplementary Table 7). Pigmentation-related genes such as *TYR* and *TSPAN10* surfaced across multiple foveal traits, underscoring the role of melanin synthesis in the shared developmental pathways that shape foveal morphology (Table 1). This is in keeping with previous observations in albinism^33,34^ and in a smaller cohort where a link between the common functional *TYR*:c.1205G>A (p.Arg402Gln) [rs1126809] variant and decreased rim radius was reported.^35^

While many of the lead loci detected here have previously been linked to retinal traits, at least 124 of them have no such prior associations (Supplementary Dataset S1). Two examples are *CYP1A1* and *PPP1R9A. CYP1A1* (lead marker: rs200391170; association with mean slope; p = 2.2 × 10^−21^) encodes a major cytochrome P450 enzyme and is involved in the metabolism of both endogenous substances and environmental chemicals. It has been implicated in the conversion of all-trans retinol to all-trans retinoic acid in human fetal tissues.^36^ Notably, retinoic acid gradients are essential for dorsoventral patterning, and retinoic acid signaling has been directly linked to the positioning of the fovea-like area in the chick retina.^37,38^ Another gene that has not been previously linked to retinal phenotypes is *PPP1R9A* (lead marker: rs9649194; association with pit depth and mean slope; minimum p = 6.5 × 10^−14^). This gene encodes a protein phosphatase that modulates actin cytoskeleton organization,^39^ and one possibility is that it plays a role in the formation of the foveal pit (rather than the patterning/positioning of this high-acuity structure to the correct retinal region, a process that occurs earlier in development).

A subset of the detected lead loci (n=36) has been previously linked to retinal thickness measurements (either clinically-used^15–17^ or deep learning based^5,19,40^) but not with foveal/macular traits (Supplementary Dataset S1). These novel associations that we report here enhance the interpretability of the signals and allow more robust links to be drawn with central retinal development/patterning. Two notable examples are *FGFR2* and *PTPRD. FGFR2* (lead marker: rs146727842; association with mean slope; p = 1.4 × 10^−20^) encodes a receptor tyrosine kinase that is essential for embryonic development.^41^ *FGFR2* has been shown to interact with *FGF8*, whose expression is required to pattern the chick fovea-like area.^37,38,42^ *PTPRD* (lead marker: rs7020703; association with rim radius; p = 1.3 × 10^−37^) encodes a tyrosine phosphatase that regulates neural development by modulating receptor tyrosine kinase activity.^16,43^ A related protein (cRPTPlambda) is found to be absent from the chick fovea-like area, and it can be speculated that *PTPRD* is involved in regulating FGF signaling early to set up the foveal region.^44^

This study has several limitations. First, obtaining accurate quantitative measurements of OCT-derived retinal features is inherently challenging. One issue is image scaling, as precise measurements require knowledge of the exact lateral scale of each scan. To address lateral magnification, we included spherical equivalent refractive error as a covariate in our analyses. While this method is less accurate than using ocular axial length^31,48^, such measurements are not available in the UK Biobank dataset. It is noted though that our findings showed good agreement with those from smaller, more targeted studies.^47,49^ This approach is also consistent with most previous genetic association studies involving retinal imaging-derived phenotypes.^15,16,18–20^ Second, we relied on UK Biobank healthcare record data to identify cases of AMD and glaucoma. These records are known to be incomplete, and several alternative methods using retinal imaging data have been proposed.^50,51^ Although these have shown positive diagnostic performance, they can overestimate prevalence,^51^ and we opted to use a conventional approach focused on record-based diagnoses. Finally, while the UK Biobank was designed to be broadly representative of the ageing UK population, it is subject to selection biases. All reported associations should therefore be interpreted considering this limitation.^52,53^

In conclusion, we show that (i) foveal architecture is patterned by sex, genetic ancestry and genes related to pigmentation, and (ii) one aspect of that architecture, pit volume, appears to lie on the causal pathway to AMD. These findings position foveal pit metrics as potential biomarkers of macular vulnerability and invite mechanistic work to dissect how developmental pathways intersect with ageing to drive retinal degeneration.

## MATERIALS AND METHODS

### Ethical approval

The UK Biobank has received approval from the UK National Information Governance Board for Health and Social Care and the National Health Service North West Centre for Research Ethics Committee (Ref: 11/NW/0382). This research was conducted using the UK Biobank Resource under projects 53144 and 49978. All investigations were conducted in accordance with the tenets of the Declaration of Helsinki.

### UK Biobank: cohort characteristics and ophthalmic phenotyping

We used data from the UK Biobank, a resource containing genomic and health information from 502,355 individuals from across the United Kingdom, aged between 40 and 69 years at the time of recruitment.^3^ Subsets of UK Biobank volunteers underwent enhanced phenotyping including visual acuity testing (>130,000 individuals), non-cycloplegic autorefraction (>125,000 individuals) and imaging of the central retina using color fundus photography and OCT (>84,000 individuals). Baseline ophthalmic assessment was conducted between 2009 and 2010 for most participants and between 2012 and 2013 for a smaller subset.^4^

Best-corrected visual acuity was assessed using a computer-displayed LogMAR chart (Precision Vision, LaSalle, IL, US) (UK Biobank data-fields: 5208 and 5201). Participants wore their habitual distance correction and were tested at 4 m; if unable to identify any letters, the chart was moved to 1 m. Starting from the top line, testing stopped once ≥2 letters on a line were misread.^4^

Non-cycloplegic autorefraction was carried out using a Tomey RC 5000 device (Tomey Corp., Nagoya, Japan). The spherical equivalent refractive error (spherical error + 0.5 × cylindrical error) was subsequently calculated for each participant (data-fields 5084–5085; 5086–5087).^4^

Color fundus photographs and spectral-domain OCT images were acquired using the Topcon 3D-OCT 1000 Mark II device (Topcon Corp., Tokyo, Japan), without pharmacological dilation (data-fields 21015, 21016, 21011, 21013). OCTs were obtained using a ‘3D macular volume’ protocol (6 × 6 mm; 128 horizontal B-scans, each comprising 512 A-scans arranged in a raster pattern).^5,40,54^ The right eye was imaged first. Our analysis focused on left eye images as we assumed that familiarity with the test would have led to scans that, on average, had higher overall quality.^5,18^

### Fundus pigmentation quantification

We used a recently described method^21^ to evaluate fundus pigmentation in UK Biobank participants. This involved calculating the Retinal Pigment Score (RPS), a continuous quantitative metric that is thought to primarily reflect choroidal pigmentation.^55^

RPS was derived using the approach described by Rajesh *et al*. which entails selecting fundus photographs of sufficient quality (*i.e*. those labeled as ‘Good’ by Automorph, an established deep learning tool)^56^ and processing them through the RPS pipeline to quantify background pigmentation.^17^

### OCT quality control and foveal morphological parameter extraction

OCT scans were filtered for overall quality in line with previous studies.^18,20^ Briefly, we discarded OCTs that (j) had a quality score below 40 and (ii) fell within the lowest 10% of the inner limiting membrane (ILM) edge-strength indicator (a metric that captures the sharpness and continuity of the ILM boundary and flags blinks, signal dropouts, and major segmentation errors).^20^

OCT images meeting the above quality control criteria were processed using RETIMAT, an open-source MATLAB toolbox for OCT image analysis. The processing pipeline included the following steps:

- parsing Topcon (.fda) files to extract the ILM and Bruch’s membrane (BM) surfaces delineated by the device software;
- computing total retinal thickness maps;
- automatically aligning thickness maps to the foveal center;
- transforming raster thickness maps into a radial pattern; and
- computing morphometric values.

RETIMAT has been used successfully in previous studies and its fully automated methods for fovea location and analysis have been independently assessed, making it well-suited for high-throughput analysis.^14,57^ We focused on six RETIMAT-derived foveal traits: foveal pit volume, rim radius, rim height, mean slope, pit depth and central foveal thickness (CFT) (Fig.1, Table 2).

Aiming to study the phenotypic variability of these parameters, we assembled a broad ‘phenotypic analysis’ cohort. This included UK Biobank participants whose OCT scans passed the imaging quality control criteria for this study; whose RETIMAT-derived foveal trait values did not fall in the 1% upper or lower extreme; and who were assigned to one of the four main genetically-inferred ancestral groups (EUR, AFR, SEA, and SAS; see ‘Genetic ancestry assignment’ section below). Aiming to study the genetic architecture of foveal traits, a second, more focused ‘genetic analysis’ cohort was defined. This was limited to unrelated participants of European-like ancestries with OCT scans; no sex chromosome aneuploidy; and not flagged as outliers for heterozygosity nor missingness. Individuals with RETIMAT-derived foveal trait values exceeding 2.5 standard deviations were also excluded (Supplementary Fig.S15); this cut-off was selected in accordance with the approach used in previous genetic association studies of retinal thickness measurements.^15,16,18–20^ The final GWAS sample comprised 36,205 unrelated individuals with European-like ancestries (29,710 primary plus 6,495 replication); the broader phenotypic cohort totaled 55,604 individuals across ancestries.

### Genetic ancestry assignment

To facilitate inter-ancestry analyses of foveal traits, we sought to characterize the genetic ancestry of UK Biobank participants. A subset of 409,373 participants with European-like ancestries is readily-available (data-field 22006) and we aimed to obtain genetic ancestry estimates for the remaining 78,296 individuals. Using a previously-described approach^58^ we projected these UK Biobank participants onto reference genetic data from the 1000 Genomes Project.^59^ In brief, we used directly genotyped single-nucleotide variants from the UK Biobank and genetic data from the 1000 Genomes Project (Phase 3, v5a.20130502). After matching variants between these two resources, we merged the two datasets using PLINK v2.0,^60^ retaining 718,487 high-quality variants. We then performed linkage disequilibrium (LD) pruning and removed regions with long-range LD, resulting in 30,320 independent variants. Ancestry proportions were estimated using ADMIXTURE (v1.3.0),^61^ with four reference groups from the 1000 Genomes Project dataset: GBR (European), YRI (African), ITU (South Asian), and CHS (East Asian). Only individuals with at least 80% ancestry probability assigned to one of these four groups were retained for further phenotypic analyses.

### Statistical analyses of foveal phenotypes

The relationships between foveal traits, age of OCT image acquisition and spherical equivalent refractive error were studied using Spearman’s correlation analyses. Further, comparisons were made between sex and genetic ancestry groups for each of the studied foveal traits using the Wilcoxon rank-sum test (when comparing two groups) and the Kruskal–Wallis test (when comparing more than two groups).

To quantify the contributions of fundus pigmentation (RPS) and genetic ancestry to variability in foveal morphology, we fitted linear regression models for each trait, as follows: The two predictors were analyzed together given their presumed biological relationship (as pigmentation traits vary by ancestry and may capture overlapping sources of variation).

*Model 1:* included RPS, spherical equivalent refractive error, and sex.

*Model 2* included genetic ancestry (as a categorical variable), spherical equivalent refractive error, and sex.

*Model 3*: included both RPS and genetic ancestry terms, along with spherical equivalent refractive error and sex.

From each model, we extracted adjusted R^2^ values to assess explained variance. Unique contributions of RPS and ancestry were derived by comparing the full model against models excluding one predictor set at a time.

Finally, the association between foveal morphology and visual acuity was studied using a linear regression approach with age, sex, spherical equivalent refractive error and genetic ancestry used as covariates.

### Genome-wide association studies (GWAS)

We performed a common-variant GWAS using an additive linear model in REGENIE v3.1.1.^22^ To meet the normality assumptions of GWAS, we applied rank-based inverse normal transformation to any foveal traits that deviated from a normal distribution. For this analysis, we focused only on individuals of European-like genetic ancestries (data-field 22006). We performed a primary and a replication GWAS. The focus of the former was on the 67,250 individuals that were imaged at the time of their baseline visit (Instance 0, ‘Initial assessment visit (2006–2010)’). The focus of the replication study was on the 15,568 different participants that were imaged for the first time during their first repeat assessment (Instance 1, ‘First repeat assessment visit (2012–2013)’).

To process the imputed genotype data, we applied conventional quality control filters using PLINK2.^60^ Variants were retained if they had: an imputation quality score above 0.8; a minor allele frequency (MAF) of 5% or more; and a minor allele count (MAC) of 20 or more. Variants with a Hardy-Weinberg equilibrium p-value below 1 × 10^−15^ were excluded, along with those with missingness greater than 10%. Individuals with more than 10% missing genotypes were also excluded.^22^

The GWAS model was adjusted for age at recruitment (data-field 21022), sex (data-field 31), height (data-field 50), weight (data-field 21002), spherical equivalent refractive error (calculated from data-fields 5085 and 5086 and defined as spherical error + 0.5 × cylindrical error), and the first 20 genetic principal components (data-field 22009). Genome-wide statistical significance was set at p < 5 × 10^−8^.

To refine the obtained association signals, further analyses were performed using the GCTA-COJO tool (conditional and joint multiple-variant analysis).^23^ Variants located on different chromosomes, or more than 10 Mb apart, were treated as unlinked. Lead genetic variants were then annotated using Ensembl,^62^ Open Targets,^63^ and GWAS Catalog^64^ data (accessed on June 24, 2025). To accurately summarize the strongest signals, the LD metrics of the changes that were highlighted as lead variants by GCTA-COJO analysis and were within 1 Mb of one another were manually inspected using the LDlink tool.^65^

### LD score regression: heritability estimation and global genetic correlation analysis

The global genetic correlations between the six studied foveal traits (pit volume, rim radius, rim height, mean slope, pit depth, and CFT) and RPS were calculated using LD score regression.^66^ The GWAS summary statistics for RPS were obtained from a study by Julien *at al*.^55^ The analysis adhered closely to the methodology described by Bulik-Sullivan *et al*.,^66^ with genetic variants filtered according to the following criteria: inclusion in HapMap3; minor allele frequency > 0.01; imputation score > 0.9, exclusion of strand-ambiguous variants; and removal of duplicated variants. LD scores were derived from the European subset of the 1000 Genomes Project dataset.^59^

LD score regression was also used to calculate heritability, λ_G_ (genomic inflation factor), intercept, and ratio (denoting the proportion of inflation resulting from confounding or bias) for each of the six studied foveal traits.

### Cox regression analysis

We performed Cox proportional hazards regression to investigate the association between foveal traits and incident age-related macular degeneration (AMD) and glaucoma. Participants with available OCT-derived foveal traits and outcome data were included in the analysis (n = 55,604). Disease status was defined using hospital inpatient ICD-10 diagnosis fields (data-field: 41202; ICD-10 H35 and H40). The date of baseline imaging (Year of Scan, YOS) was extracted from UK Biobank data-field 21836 and used to define time-zero for each participant.

Age at scan was computed from YOS and the month and year of birth for each participant. We defined disease onset as the earliest recorded date for the relevant ICD-10 code. Participants were censored at the last available follow-up date (May 31, 2022), if their imaging date was missing, or if disease onset occurred before the scan.

Cox regression models were fitted using the ‘lifelines’ Python package,^67^ with time-to-event (in days) as the outcome, and foveal traits as the predictors. All models were adjusted for age at image acquisition, sex, spherical equivalent refractive error, and genetic ancestry. Ancestry was modelled using one-hot encoded genetic ancestry categories with individuals of European-like ancestries as the reference group. Models were fit for each trait–disease pair. Hazard ratios (HR), 95% confidence intervals, and p-values were extracted. The p-values were corrected for multiple testing using the Benjamini–Hochberg false discovery rate (FDR) method.

To enhance the interpretability of the detected associations, we performed logistic regression using a binary outcome defined as the presence or absence of disease onset after the scan date and before the censoring date (May 31, 2022). Individuals who had not developed the disease by the censoring date were treated as controls. Covariates included age at image acquisition, sex, spherical equivalent refractive error, and genetic ancestry (encoded as dummy variables). Analyses were run separately for each foveal trait. Logistic regression models were fitted using the ‘statsmodels’ Logit function in Python^68^, and odds ratios (with 95% confidence intervals) and p-values were extracted.

### Causal inference using two-sample Mendelian randomization

Causal associations between AMD, glaucoma and the six studied foveal traits were assessed using univariable two-sample Mendelian randomization (MR).^69,70^ Population overlap biases univariable two-sample MR in the direction of causation. Accordingly, the populations contributing toward traits determined to be causal were inspected to ensure that there was no overlap. Genetic instruments were selected based on p < 5 × 10^−8^ in relation to each exposure trait. Where an exposure instrument was not present in the outcome dataset, we sought to identify a suitable proxy. Proxy variants were single-nucleotide variants that were in high LD with exposure-associated variants which were not present in the outcome dataset. Proxies were identified using the Ensembl server,^62^ selecting variants with an LD R^2^ value of 0.9 or above. Genetic variants were clumped using an LD R^2^ value of 0.001 and a genetic distance cut-off of 10,000 Kb. This was achieved using the 1000 Genomes Project European superpopulation reference panel and the TwoSampleMR PLINK clumping function.^59,60^ The effects of instruments on outcomes and exposures were harmonized to ensure that the β values (*i.e*. the regression analysis estimates of effect size) were signed with respect to the same alleles. For palindromic alleles (*i.e*. alleles that are the same on the forward as on the reverse strand), those with minor allele frequency (MAF) > 0.42 were omitted from the analysis to reduce the risk of errors due to strand issues. In addition to using a range of Mendelian randomization methods and quality control measures (as detailed below), we endeavored to remove pleiotropic instruments and outliers from the analysis. To achieve this, instruments that were more statistically significant (in terms of p-value) for the outcome than the exposure were removed. Radial Mendelian randomization,^71^ an approach that detects outlying instruments, was also used for outlier identification, and outliers were subsequently removed.^71^ The Cochran’s Q test was performed for each analysis. The MR Egger intercept^72^ was used to detect horizontal pleiotropy. The I^2^ statistic was calculated as a measure of heterogeneity between variant specific causal estimates. An I^2^ < 0.9 indicates that MR Egger is more likely to be biased towards the null through violation of the “NO Measurement Error” (NOME) assumption. Our primary analysis was a multiplicative random effects inverse variance weighted (IVW) test, which is the most efficient analysis method with valid instrumental variables.^73^ It is noted that Mendelian randomization relies upon several assumptions holding true to provide an accurate assessment of causation. The core assumptions are that (i) the instrumental variables (single-nucleotide polymorphisms) utilized are associated with the exposure; (ii) the instrumental variables are not associated with the outcome via a confounder; and (iii) the instrumental variables do not impact the outcome other than through the exposure being considered (*i.e*. no horizontal pleiotropy).^73^ Robust analyses are those which can provide valid causal inference under weaker assumptions than an IVW test, and are important for a complete Mendelian randomization study. Robust tests included the weighted median (robust to up to 50% of invalid instruments), weighted mode (robust to >50% of invalid instruments but assumes more variants estimate the true causal effect than estimate any other quantity), MR Egger (robust to pleiotropic effects of variants, provided the effects are independent of the variant-exposure associations) and a leave-one-out analysis using the MRE IVW method (to test for highly influential variants).^73^ When appraising the results of a Mendelian randomization study, the confidence with which one can assert causality is influenced by both the strength of association in the primary analysis (IVW) and the range of robust tests in which the results are consistent. Where the IVW is significant but robust tests are not, this may be in keeping with causality, but under stricter assumptions of validity across the instrumental variables utilized (and therefore may warrant more cautious interpretation).

## Supplementary Material

Supplement 1

Supplement 2

## Figures and Tables

**Table 1.** Summary of the 10 top-ranking loci associated with foveal traits.

Top-ranking common variant in locus	Chr: position (GRCh37)	Key gene(s)	Allele freq	Foveal trait with significant result (minimum p-value)	Selected previous associations in the GWAS Catalog; [Panelapp association(s)]
rs6420484	17:79612397	*TSPAN10 / NPLOC4 / PDE6G*	0.65	Pit depth (2.01 × 10^−107^); Pit volume (7.41 × 10^−105^); Rim radius (3.54 × 10^−59^); Mean slope (3.16 × 10^−55^); Rim height (4.88 × 10^−48^)	Retinal thickness measurements, refractive error, eye color, hair color
rs3138142	12:56115585	*RDH5* / *CD63*	0.24	Rim height (1.98 × 10^−77^); Pit depth (2.86 × 10^−14^); Mean slope (6.21 × 10^−13^)	Retinal thickness measurements, refractive error, retinal vascular fractal density; [retinal dystrophy]
rs11708067	3:123065778	*ADCY5*	0.24	Rim radius (6.34 × 10^−69^); Pit volume (7.95 × 10^−56^)	Retinal thickness measurements, height, birth weight, type 2 diabetes, glucose levels, HbA1c levels, cholesterol levels; [dyskinesia]
rs1042602	11:88911696	*TYR*	0.37	Pit depth (1.67 × 10^−62^); Pit volume (8.26 × 10^−58^); Rim radius (1.16 × 10^−22^; Mean slope (3.48 × 10^−39^); CFT (9.32 × 10^−40^);	Retinal thickness measurements, brain measurements, skin color, hair color; [albinism]
rs9298817	9:21576591	*MIR31HG*	0.66	Pit volume (1.43 × 10^−61^); Rim radius (1.44 × 10^−21^); Rim height (2.30 × 10^−20^)	Retinal thickness measurements, thyroid medication use
rs17421627	5:87847586	*LINC00461*	0.07	Pit volume (3.12 × 10^−46^); CFT (1.34 × 10^−35^); Rim radius (3.87 × 10^−32^); Rim height (1.60 × 10^−27^)	Retinal thickness measurements, retinal vascular fractal density
rs11051145	12:31050201	*TSPAN11*	0.16	Pit volume (5.02 × 10^−46^); CFT (5.22 × 10^−34^)	Retinal thickness measurements, optic disc size
rs7020703	9:8632149	*PTPRD*	0.22	Rim radius (1.25 × 10^−37^)	Retinal thickness measurements, bone mineral density, height, restless leg syndrome
rs67138036	4:187683202	*FAT1*	0.26	Rim radius (4.58 × 10^−37^)	Retinal thickness measurements; [microphthalmia/coloboma, nephropathy]
rs1493523	15:44345614	*CCDC122*	0.68	Pit volume (1.86 ×10^−36^)	-

CFT, central foveal thickness

**Table 2.** Definitions of the six RETIMAT-derived foveal traits analysed in this study

Foveal trait	Definition
Pit volume (mm^3^)	Integrated volume between rim height and the full thickness profile
Rim radius (mm)	Radial distance from center to point of rim height
Rim height (μm)	Maximum thickness at the foveal rim
Mean slope (°)	Average inclination from center to rim
Pit depth (μm)	Rim height minus central foveal thickness
Central foveal thickness (μm)	Thickness at the foveal center

A pictorial representation of rim radius, rim height and mean slope can be found in Fig.1

## Data Availability

UK Biobank data are available under restricted access through a procedure described at http://www.ukbiobank.ac.uk/using-the-resource/. All other data supporting the findings of this study are available within the article (including its Supplementary Information files).

## CODE AVAILABILITY

The scripts used to analyse the datasets included in this study are available at https://github.com/davidjohngreen/fovea and https://github.com/mubiomed/retimat.

## UK Biobank Eye and Vision Consortium

Jay E. Self,^9,10^ Graeme C. Black^1,11^, Panagiotis I. Sergouniotis^1,3,5,11^

^1^ Division of Evolution, Infection and Genomics, School of Biological Sciences, Faculty of Biology, Medicine and Health, University of Manchester, Manchester, UK.

^3^ Manchester Royal Eye Hospital, Manchester University NHS Foundation Trust, Manchester, UK.

^9^Clinical and Experimental Sciences, Faculty of Medicine, University of Southampton, Southampton, UK

^10^Southampton Eye Unit, University Hospital Southampton NHS Foundation Trust, Southampton, UK

^11^Manchester Centre for Genomic Medicine, Saint Mary’s Hospital, Manchester University NHS Foundation Trust, Manchester, UK

A full list of members appears in the Supplementary Information.
