# Supplementary material for: Genetic insights into foveal morphology and its associations with pigmentation and age-related macular degeneration
Source: medRxiv. 2025 Jun 28:2025.06.27.25330434. Preprint. [Version 1] doi: 10.1101/2025.06.27.25330434 (PMC12262746; doi:10.1101/2025.06.27.25330434)
Supplement: Supplement 2 [file media-2.pdf]

SUPPLEMENTARY MATERIAL

TITLE

Genetic insights into foveal morphology and its associations with pigmentation and age-related macular degeneration

AUTHOR LIST & AFFILIATIONS

David J. Green[1], David Romero-Bascones[2], Thomas H. Julian[1,3-5], Sofia Torchia[5], Heer N.V. Joisher[6-8], UK Biobank Eye and Vision Consortium, Unai Ayala[2], Maitane Barrenechea[2], Jay E. Self[9,10], Graeme C. Black[1,11], Tomas Fitzgerald[5], Ewan Birney[5], Constance L. Cepko[6-8], Joseph Carroll[12,13], Panagiotis I. Sergouniotis[1,3,5,11]

[1] Division of Evolution, Infection and Genomics, School of Biological Sciences, Faculty of Biology, Medicine and Health, University of Manchester, Manchester, UK.

[2] Biomedical Engineering Department, Faculty of Engineering (MU-ENG), Mondragon Unibertsitatea, Mondragón, Spain.

[3] Manchester Royal Eye Hospital, Manchester University NHS Foundation Trust, Manchester, UK.

[4] Christabel Pankhurst Institute, The University of Manchester, Manchester, UK

[5] European Molecular Biology Laboratory, European Bioinformatics Institute (EMBL-EBI), Wellcome Genome Campus, Cambridge, UK

[6] Department of Genetics, Blavatnik Institute, Boston, MA, USA

[7] Department of Ophthalmology, Harvard Medical School, Boston, MA, USA

[8] Howard Hughes Medical Institute, Chevy Chase, MD, USA

[9] Clinical and Experimental Sciences, Faculty of Medicine, University of Southampton, Southampton, UK

[10] Southampton Eye Unit, University Hospital Southampton NHS Foundation Trust, Southampton, UK

[11] Manchester Centre for Genomic Medicine, Saint Mary's Hospital, Manchester University NHS Foundation Trust, Manchester, UK

[12] Cell Biology, Neurobiology and Anatomy, Medical College of Wisconsin, Milwaukee, WI, USA

[13] Ophthalmology and Visual Sciences, Medical College of Wisconsin, Milwaukee, WI, USA

Correspondence to Panagiotis I. Sergouniotis (panagiotis.sergouniotis@manchester.ac.uk).

SUPPLEMENTARY FIGURES

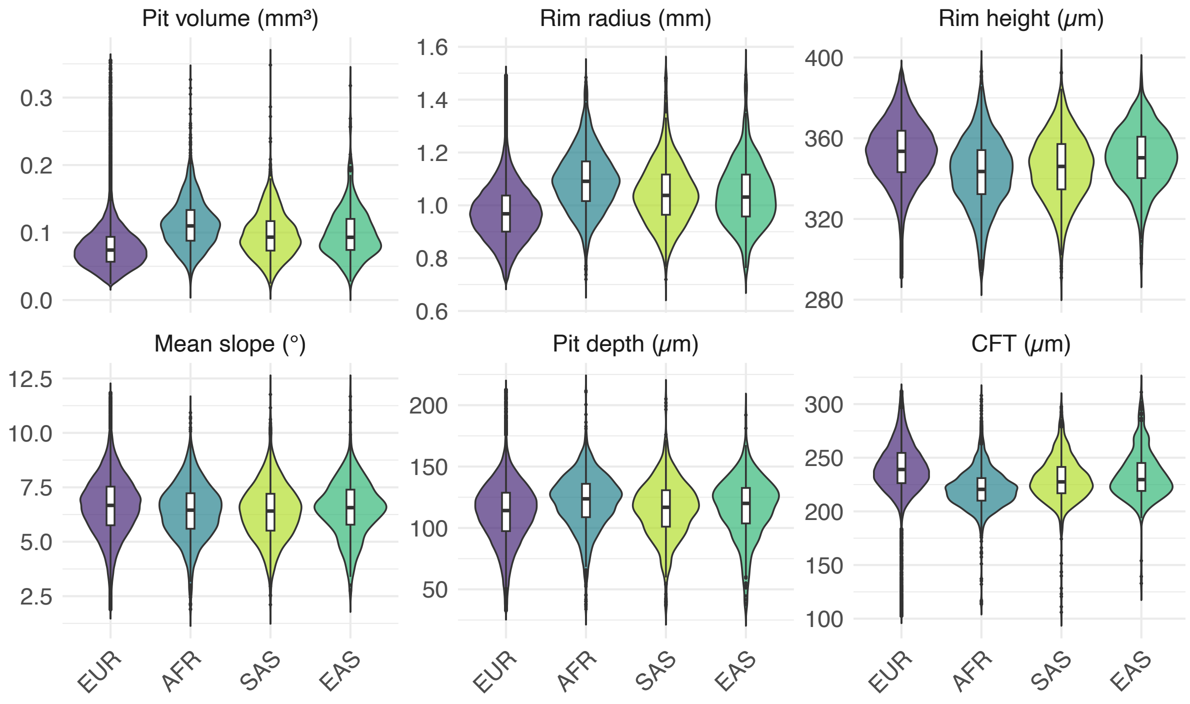

Supplementary Fig.S1. Violin plots showing the distributions of six foveal traits in individuals with European, African, South Asian and East Asian-like genetic ancestries (EUR, AFR, SAS and EAS, respectively). The Wilcoxon signed rank test was used to make comparisons and all false discovery rate-adjusted p-values were found to be $< 1 \times 10^{-10}$. It is noted that the units for the foveal traits differ and, as such, the scales should not be directly compared. Further information, including numerical data, can be found in Supplementary Table S4.

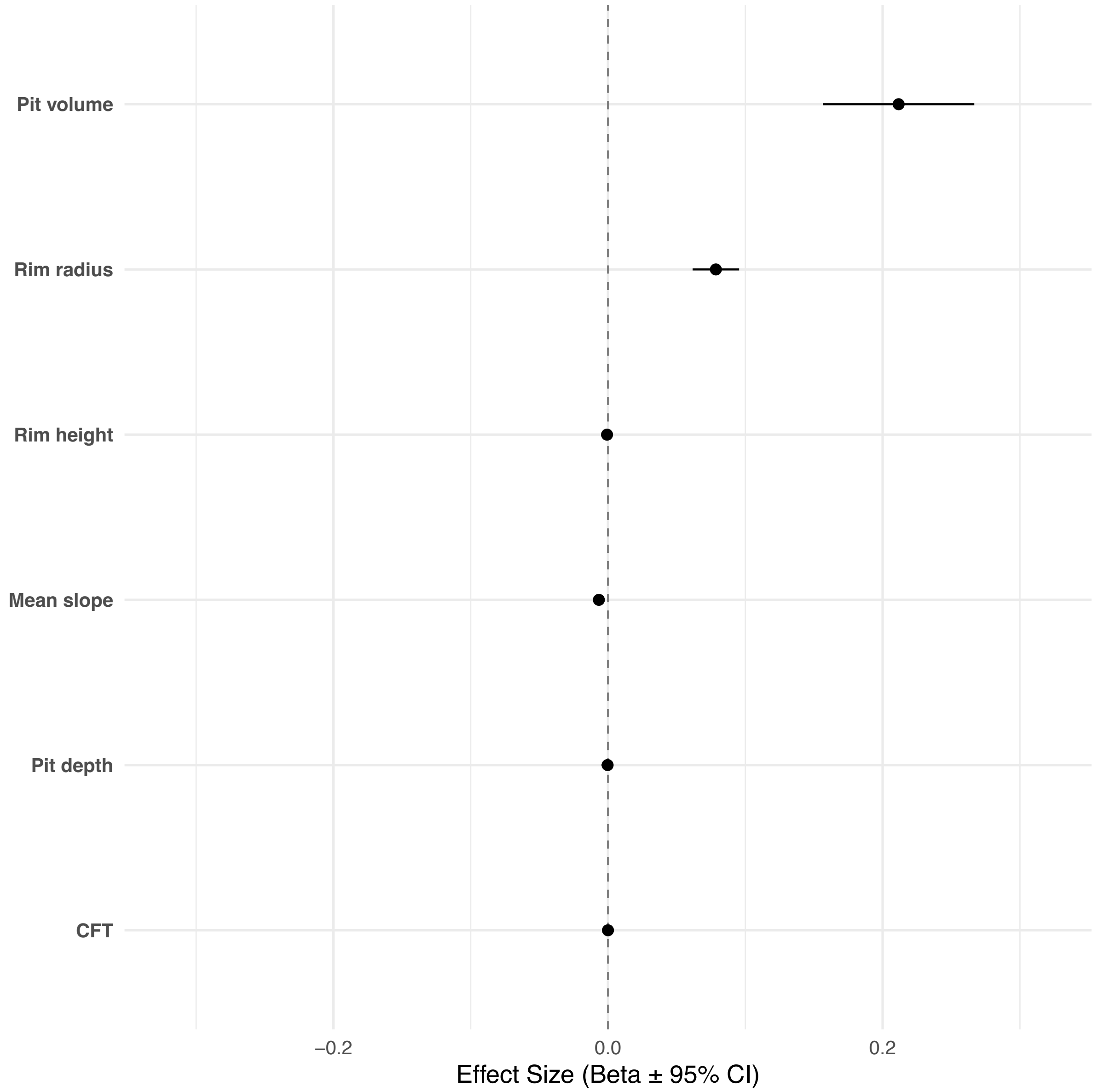

Supplementary Fig.S2. Association between foveal morphology and visual acuity. Each point shows the beta (β) coefficient (±95% CI) from a linear regression model assessing the effect of a foveal trait on visual acuity (LogMAR), adjusted for age, sex, spherical equivalent refractive error, and genetic ancestry. Negative β values indicate traits associated with better acuity. Mean slope was the strongest predictor ($\beta = -0.0081$, $p = 5.97 \times 10^{-37}$, $R^2 = 0.010$), followed by pit depth ($\beta = -0.00044$, $p = 6.03 \times 10^{-34}$), rim height ($\beta = -0.0010$, $p = 5.78 \times 10^{-71}$), and rim radius ($\beta = 0.051$, $p = 3.58 \times 10^{-9}$). Greater pit volume was linked to worse acuity ($\beta = 0.12$, $p = 4.26 \times 10^{-5}$). Central foveal thickness (CFT) showed no significant effect ($p = 0.30$). All models explained <1.3% of variance, indicating that foveal morphology is a minor contributor to visual acuity in the general population.

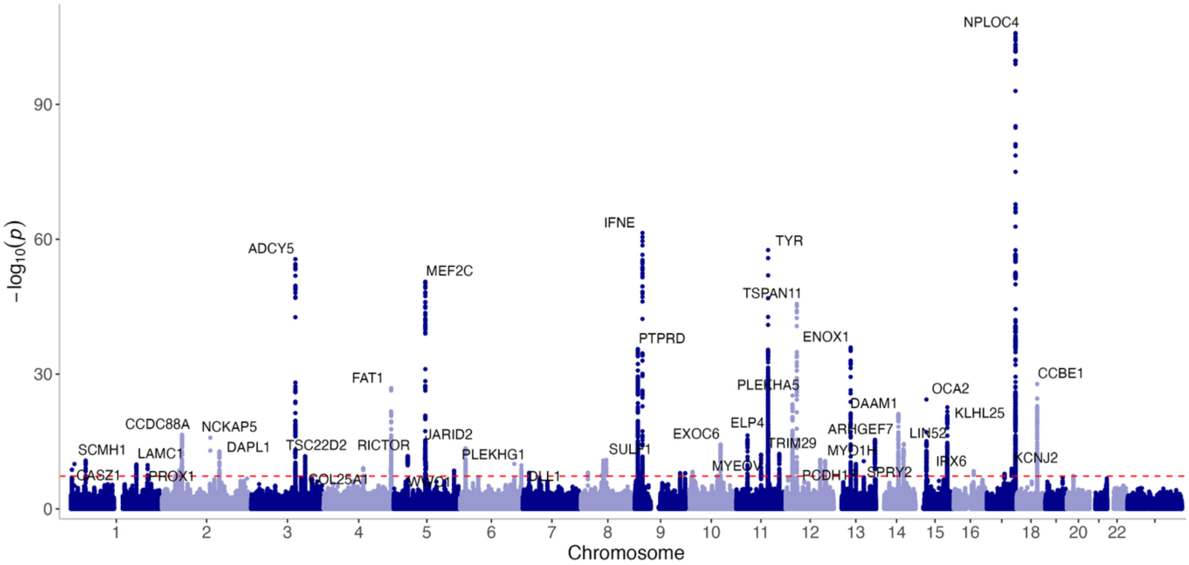

Supplementary Fig.S3. Manhattan plot showing the findings of a common-variant genome-wide association study (GWAS) for foveal pit volume. The red line indicates the threshold for genome-wide significance and key lead variants exceeding this value have been annotated.

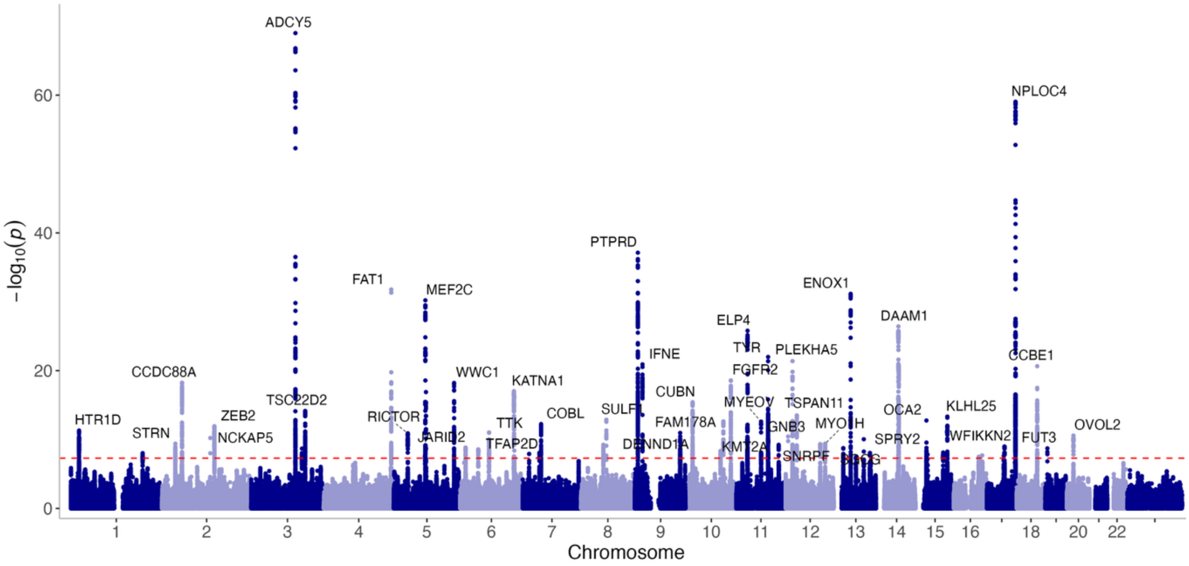

Supplementary Fig.S4. Manhattan plot showing the findings of a common-variant GWAS for foveal rim radius.

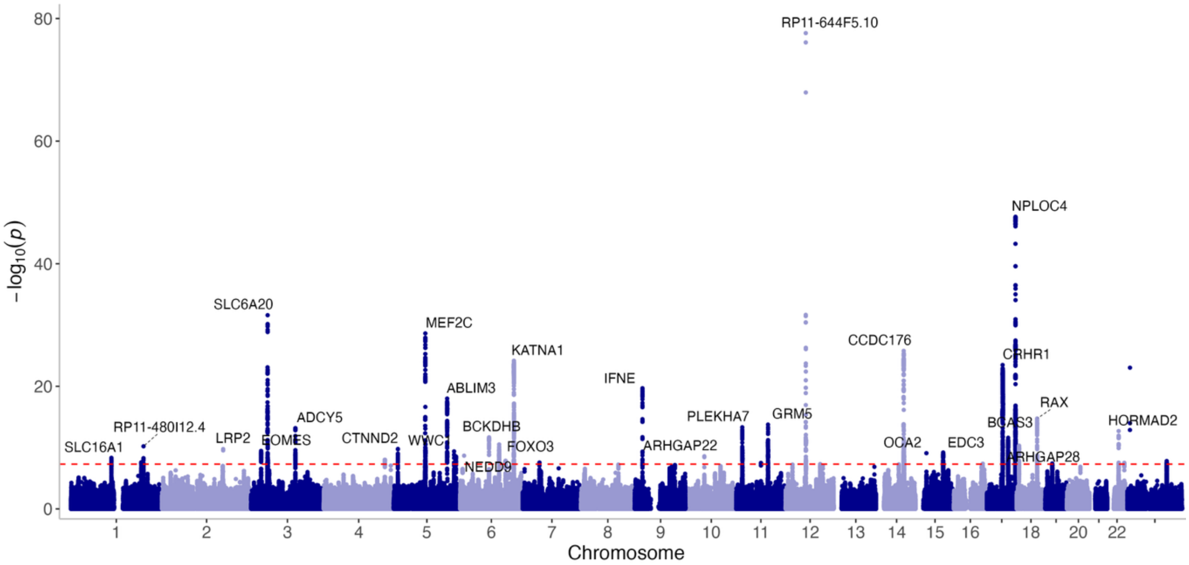

Supplementary Fig.S5. Manhattan plot showing the findings of a common-variant GWAS for foveal rim height.

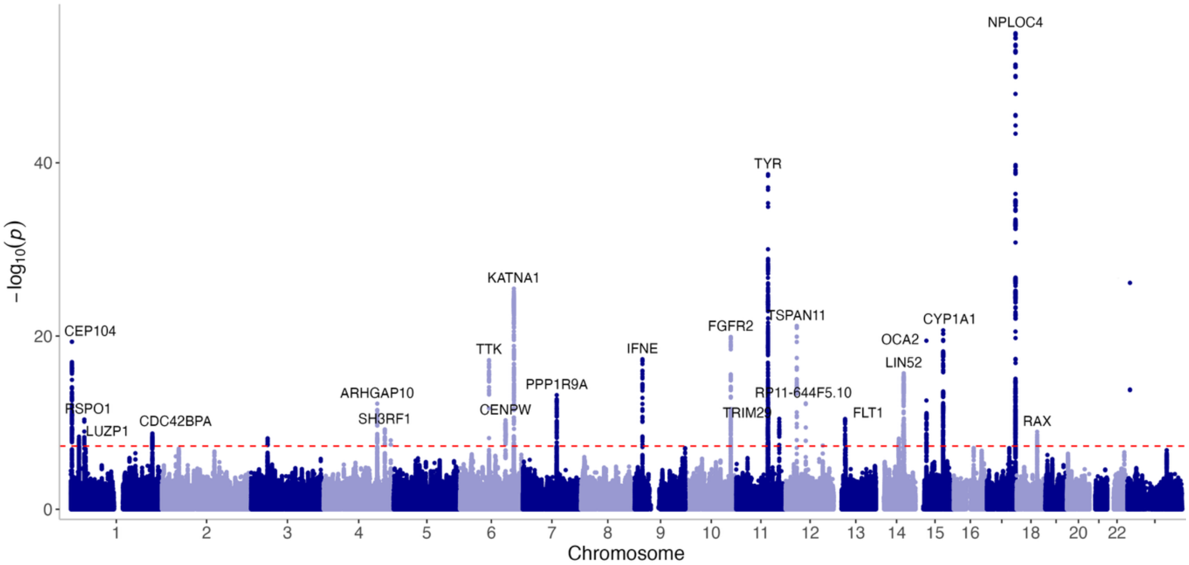

Supplementary Fig.S6. Manhattan plot showing the findings of a common-variant GWAS for foveal mean slope.

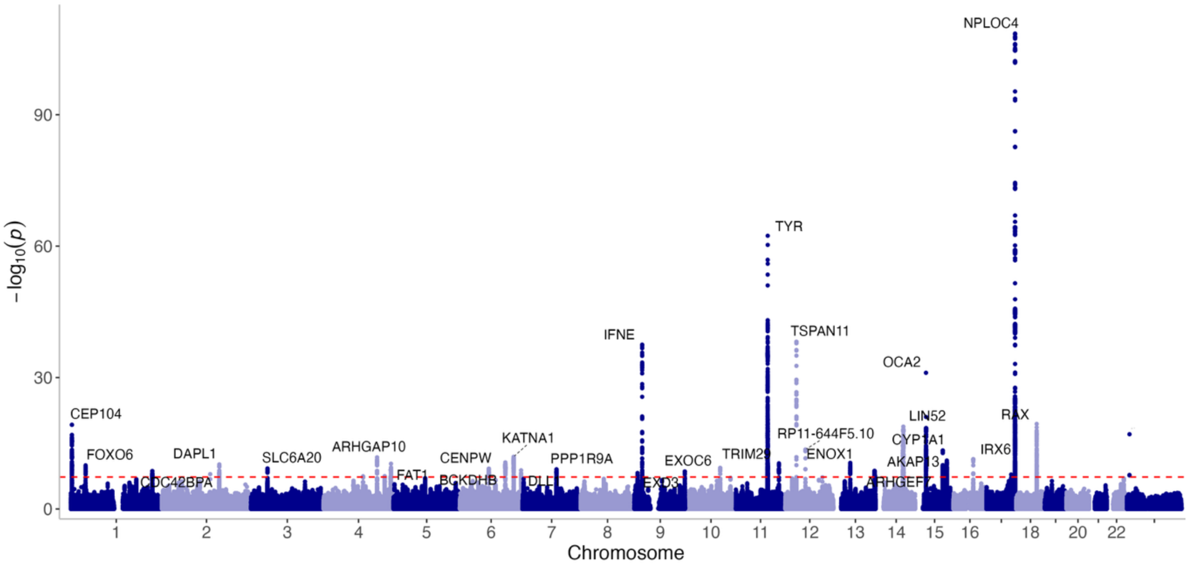

Supplementary Fig.S7. Manhattan plot showing the findings of a common-variant GWAS for foveal <u>pit depth</u>.

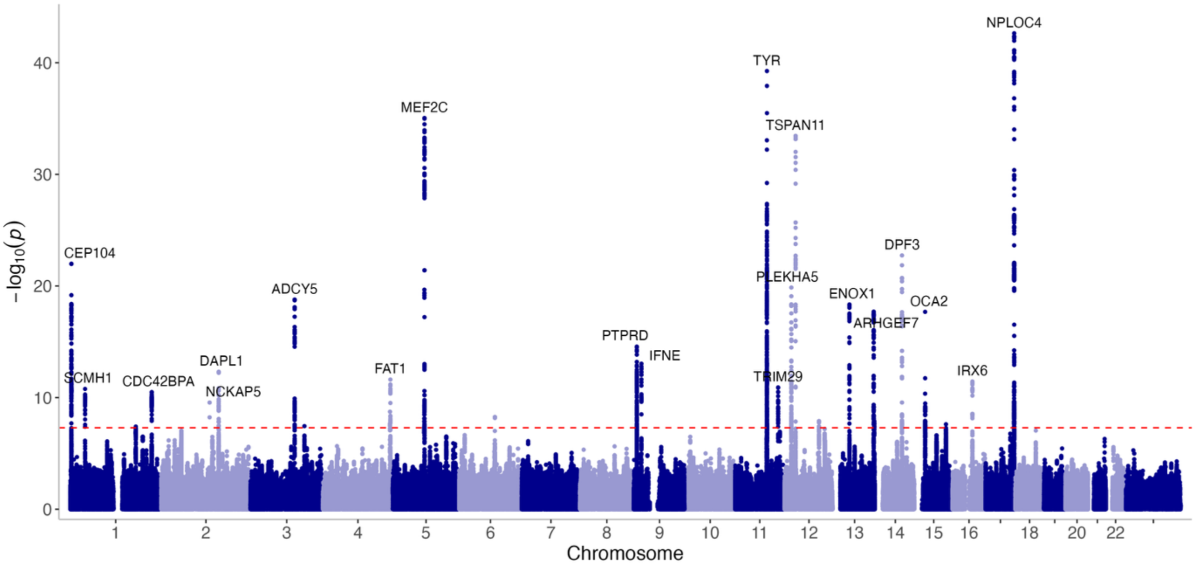

Supplementary Fig.S8. Manhattan plot showing the findings of a common-variant GWAS for <u>central foveal thickness</u> (CFT).

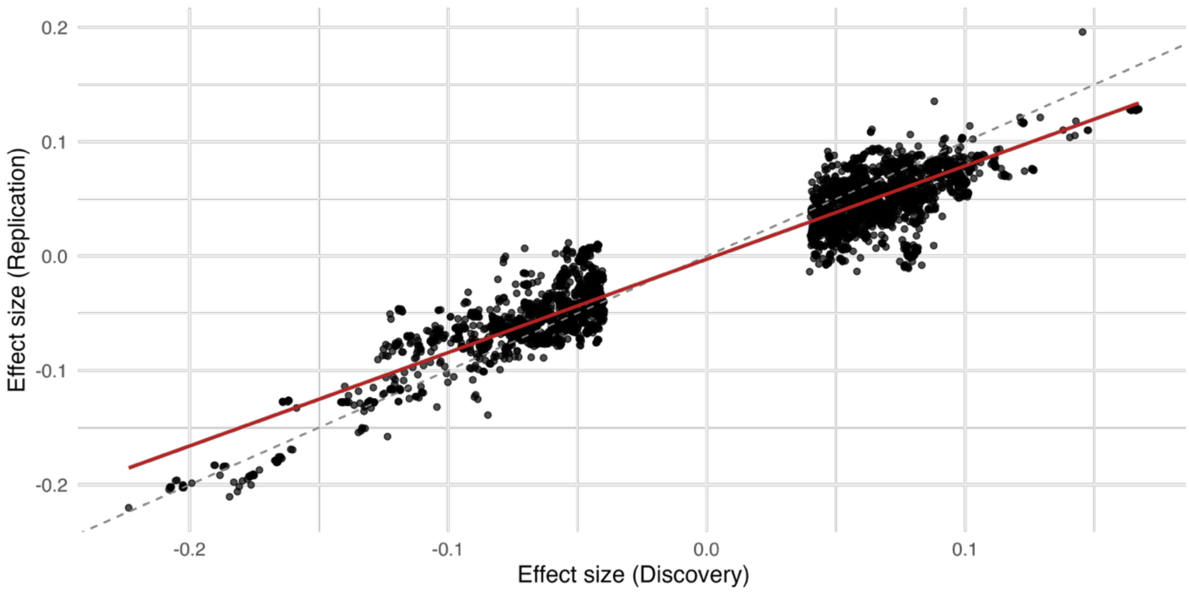

Supplementary Fig.S9. Beta–beta plot comparing effect size estimates (β) from the primary (n=29,710) and replication (n=6,495) cohorts for genome-wide significant variants associated with foveal pit volume. The dashed line indicates the identity line (y = x), and the red line shows the linear regression fit. The relevant correlation coefficient is $R^2 = 0.94$ ($p < 2.2 \times 10^{-16}$).

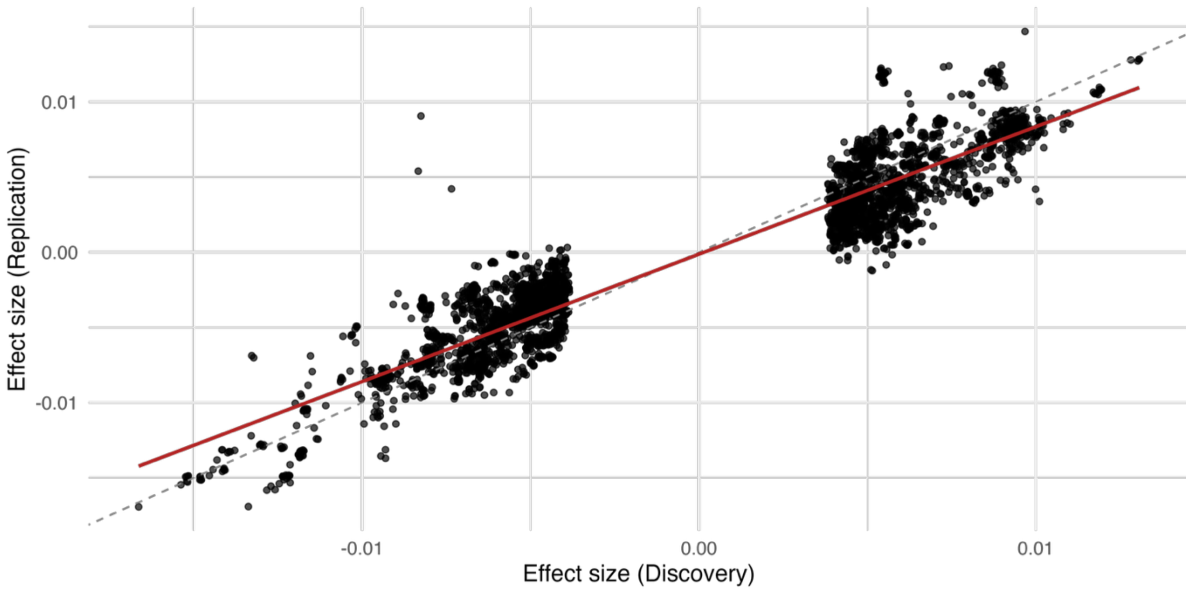

Supplementary Fig.S10. Beta–beta plot comparing effect size estimates (β) from the discovery and replication cohorts for genome-wide significant variants associated with foveal rim radius. The relevant correlation coefficient is $R^2 = 0.94$ ($p < 2.2 \times 10^{-16}$).

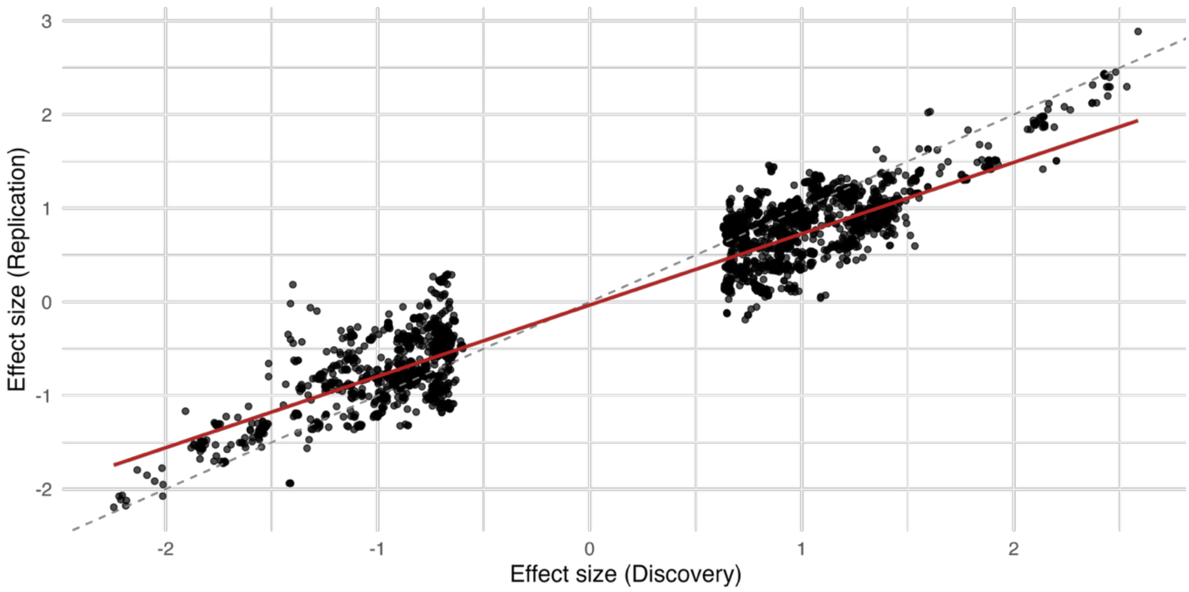

Supplementary Fig.S11. Beta–beta plot comparing effect size estimates (β) from the discovery and replication cohorts for genome-wide significant variants associated with foveal rim height. The relevant correlation coefficient is $R^2 = 0.95$ ($p < 2.2 \times 10^{-16}$).

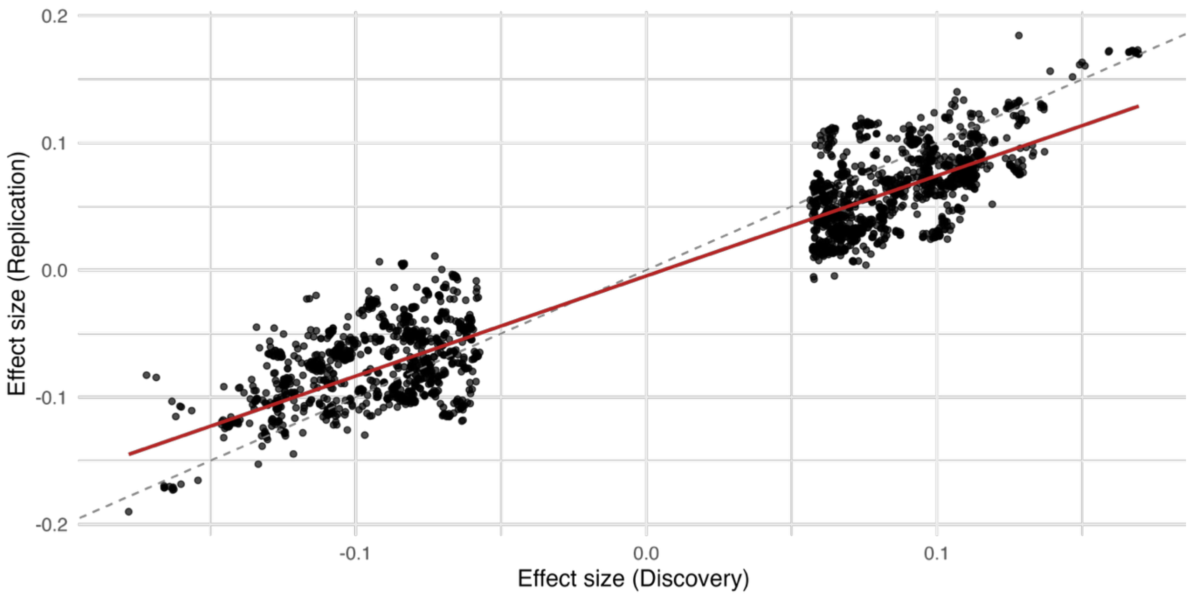

Supplementary Fig.S12. Beta–beta plot comparing effect size estimates (β) from the discovery and replication cohorts for genome-wide significant variants associated with foveal mean slope. The relevant correlation coefficient is $R^2 = 0.95$ ($p < 2.2 \times 10^{-16}$).

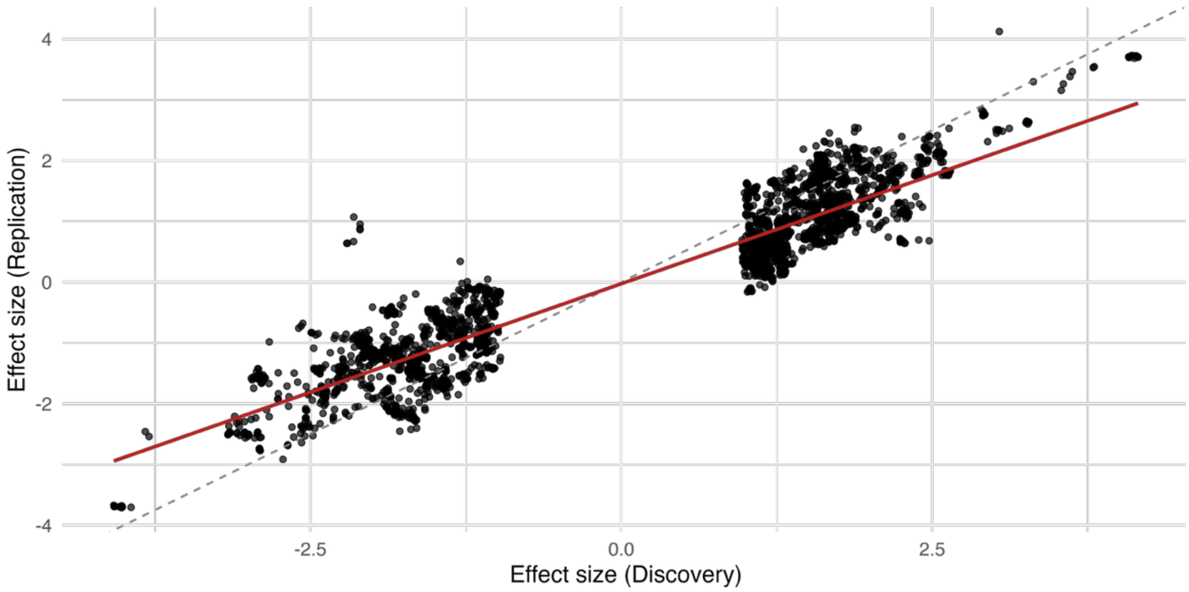

Supplementary Fig.S13. Beta–beta plot comparing effect size estimates (β) from the discovery and replication cohorts for genome-wide significant variants associated with foveal pit depth. The relevant correlation coefficient is $R^2 = 0.93$ ($p < 2.2 \times 10^{-16}$).

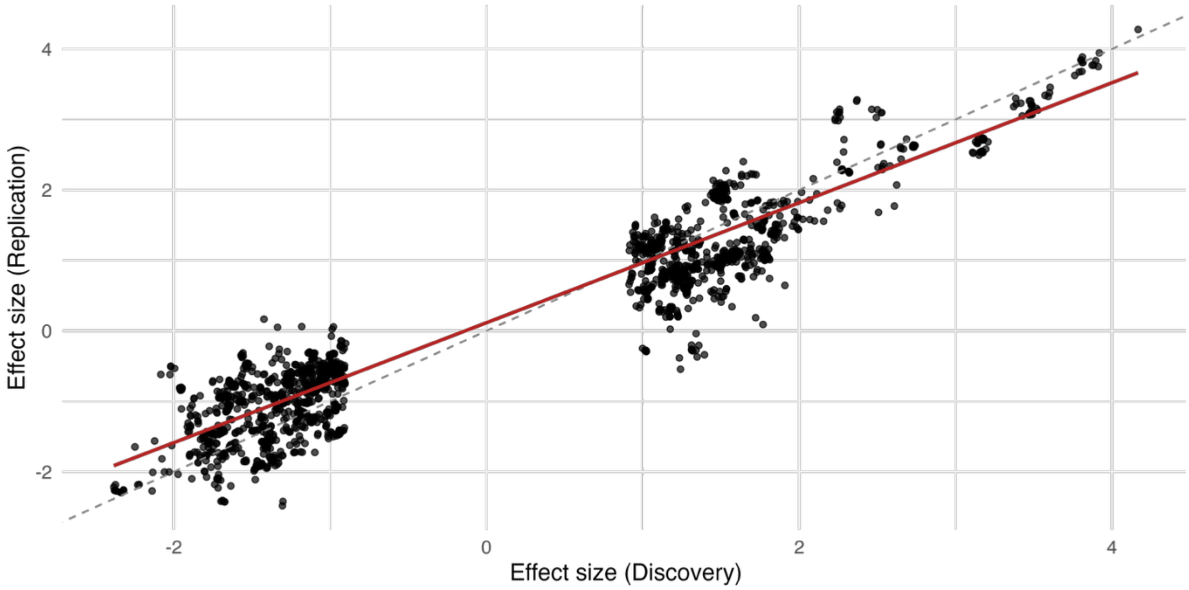

Supplementary Fig.S14. Beta–beta plot comparing effect size estimates (β) from the discovery and replication cohorts for genome-wide significant variants associated with central foveal thickness (CFT). Pearson correlation: $R^2 = 0.95$, $p < 2.2 \times 10^{-16}$.

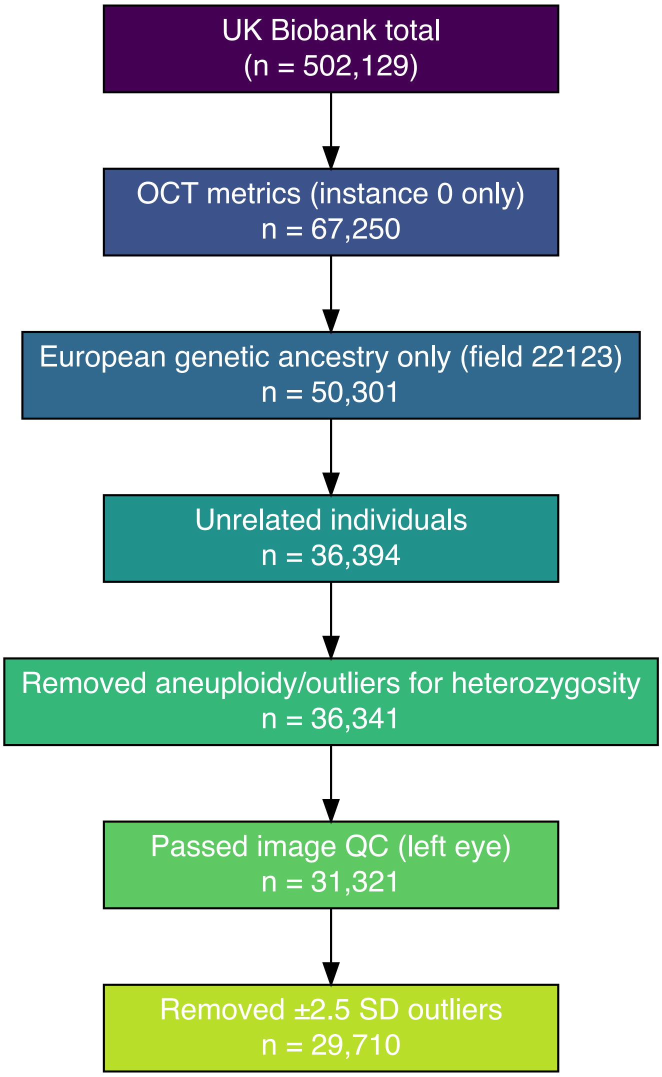

Supplementary Fig.S15: Overview of the filtering steps used in the primary common-variant GWAS. An identical approach utilizing 'instance 1' OCTs was used in the replication study**.**

OCT, optical coherence tomography; QC quality control; SD, standard deviation.

SUPPLEMENTARY TABLES

Supplementary Table S1. Correlation between foveal traits and age at OCT image acquisition

Foveal trait	Correlation coefficient	*p*-value
Pit volume	−0.118	*
Rim radius	−0.072	*
Rim height	−0.126	*
Mean slope	−0.065	*
Pit depth	−0.104	*
CFT	0.016	*

* Spearman's correlation coefficients were calculated between each foveal trait and age at OCT imaging, revealing significant but negligible associations across all traits. All p-values were $< 2 \times 10^{-4}$.

Supplementary Table S2. Correlation between foveal traits and spherical equivalent refractive error

Foveal trait	Correlation coefficient	p-value
Pit volume	0.182	*
Rim radius	0.302	*
Rim height	0.062	*
Mean slope	-0.066	*
Pit depth	0.096	*
CFT	-0.052	*

* Spearman's correlation coefficients were calculated between each foveal trait and spherical equivalent refractive error, revealing significant but variable associations across all traits. All p-values were $< 2.8 \times 10^{-33}$.

Supplementary Table S3. Differences in foveal traits between male and female UK Biobank participants

Foveal trait	Male (n=26,503)		Female (n=29,101)		p-value
	**mean**	**median**	**mean**	**median**	
Pit volume (mm^3)	0.073	0.069	0.085	0.081	*
Rim radius (mm)	0.954	0.946	1.001	0.995	*
Rim height (μm)	355.388	356.027	350.294	350.622	*
Mean slope (°)	6.694	6.758	6.538	6.574	*
Pit depth (μm)	111.277	113.044	113.712	115.651	*
CFT (μm)	243.672	242.003	236.364	234.592	*

A pictorial representation of these results can be found in Fig.3.

* All Wilcoxon rank-sum test p-values were $< 1 \times 10^{-10}$.

Supplementary Table S4. Differences in foveal traits between broad genetic ancestry groups

Foveal trait	European (n=52,640)		African (n=1,617)		East Asian (n=433)		South Asian (n=914)		p-value
	**mean**	**median**	**mean**	**median**	**mean**	**median**	**mean**	**median**	
Pit volume (mm^3)	0.078	0.074	0.114	0.110	0.100	0.093	0.098	0.093	*
Rim radius (mm)	0.973	0.968	1.094	1.091	1.042	1.031	1.046	1.037	*
Rim height (µm)	353.169	353.514	342.796	343.516	350.144	350.309	345.417	346.025	*
Mean slope (°)	6.623	6.669	6.408	6.451	6.516	6.566	6.384	6.411	*
Pit depth (µm)	112.194	114.176	121.372	123.72	116.59	119.997	115.593	116.812	*
CFT (µm)	240.649	239.044	221.264	220.5	233.65	229.638	229.309	227.5	*

A pictorial representation of these results can be found in Fig.4.

* All Kruskal–Wallis p-values were $< 1 \times 10^{-10}$

Supplementary Table S5. Results of linear models to determine the role of genetic ancestry and retinal pigment score in foveal morphology

**Trait**	**R^2** (RPS only)	**p-value** (RPS only)	**R^2** (Ancestry only)	**p-value** (Ancestry only)	**R^2** (combined)	**p-value** (combined)	**R^2** (RPS unique)	**R^2** (ancestry unique)
Pit volume	0.099	0	0.114	0	0.119	0	0.005	0.019
Rim radius	0.153	0	0.184	0	0.176	0	-0.008	0.023
Rim height	0.052	5.80 x 10^{-258}	0.069	0	0.072	0	0.003	0.020
Mean slope	0.016	5.44 x 10^{-77}	0.013	3.30 x 10^{-151}	0.019	6.48 x 10^{-88}	0.006	0.003
Pit depth	0.039	2.21 x 10^{-190}	0.029	0	0.039	3.91 x 10^{-190}	0.010	0.0005
CFT	0.038	2.23 x 10^{-187}	0.047	0	0.051	4.11 x 10^{-251}	0.004	0.013

A pictorial representation of these results can be found in Fig.5.

RPS, retinal pigment score.

Supplementary Table S6. Cox regression analysis results for associations between foveal traits and post-OCT scan incidence of AMD and glaucoma

Foveal trait	Event	Number of cases	Hazard ratio	Lower 95% CI	Upper 95% CI	p-value	p-value (FDR)
Pit volume	AMD	1225	17.406	3.536	85.674	0.000	0.001
Rim radius	AMD	1228	2.037	1.193	3.476	0.009	0.018
Rim height	AMD	1228	0.993	0.990	0.997	0.000	0.001
Mean slope	AMD	1231	0.959	0.922	0.997	0.037	0.059
Pit depth	AMD	1226	0.999	0.997	1.002	0.603	0.650
CFT	AMD	1214	0.998	0.995	1.000	0.039	0.059
Pit volume	Glaucoma	1070	0.255	0.035	1.880	0.180	0.240
Rim radius	Glaucoma	1064	0.854	0.471	1.549	0.603	0.650
Rim height	Glaucoma	1073	0.987	0.983	0.991	0.000	0.000
Mean slope	Glaucoma	1067	0.923	0.885	0.963	0.000	0.001
Pit depth	Glaucoma	1069	0.995	0.992	0.997	0.000	0.000
CFT	Glaucoma	1067	0.999	0.997	1.002	0.650	0.650

Analyses were performed in a cohort of 55,604 individuals, and all models were adjusted for age at image acquisition, sex, spherical equivalent refractive error and genetic ancestry. All numbers are rounded to 3 decimal places. A pictorial representation of these results can be found in Fig.6.

AMD, age-related macular degeneration; CI, confidence interval; FDR, false discovery rate corrected.

Supplementary Table S7. Heritability and genomic inflation estimates for the six studied foveal traits

Foveal trait	h^2	h^2 standard error	λGC	Intercept	Intercept standard error	Ratio	Ratio standard error
Pit volume	0.427	0.039	1.165	1.027	0.009	0.095	0.034
Rim radius	0.390	0.034	1.159	1.027	0.009	0.100	0.037
Rim height	0.412	0.038	1.156	1.005	0.010	0.020	0.039
Mean slope	0.311	0.036	1.120	1.014	0.009	0.065	0.047
Pit depth	0.338	0.040	1.130	1.020	0.010	0.089	0.045
CFT	0.287	0.028	1.130	1.020	0.009	0.106	0.048

SNV-based heritability (h^2) estimates were calculated using LD score regression. All traits showed moderate to high heritability, with pit volume and rim height exhibiting the highest values. The LD score intercepts remained close to 1 and attenuation ratios were low (<0.11), indicating that most inflation reflects true polygenic signal rather than confounding.

SNV, single nucleotide variant, LD, linkage disequilibrium.

Supplementary Table S8. Genetic correlation studies involving retinal pigment score and the six studied foveal traits

Trait 1	Trait 2	Correlation coefficient (rg)	Standard error	z-score	p-value
RPS	CFT	0.228	0.092	2.49	0.013
RPS	Mean slope	-0.221	0.087	-2.543	0.011
RPS	Pit depth	-0.234	0.097	-2.4	0.016
RPS	Pit volume	-0.118	0.085	-1.387	0.166
RPS	Rim height	-0.074	0.073	-1.006	0.315
RPS	Rim radius	-0.035	0.081	-0.429	0.668
CFT	RPS	0.228	0.092	2.49	0.013
CFT	Mean slope	-0.474	0.054	-8.718	2.83×10^{-18}
CFT	Pit depth	-0.693	0.038	-18.298	8.58×10^{-75}
CFT	Pit volume	-0.786	0.032	-24.557	3.61×10^{-133}
CFT	Rim height	0.198	0.070	2.833	0.005
CFT	Rim radius	-0.513	0.049	-10.492	9.36×10^{-26}
Mean slope	RPS	-0.221	0.087	-2.543	0.011
Mean slope	CFT	-0.474	0.054	-8.718	2.83×10^{-18}
Mean slope	Pit depth	0.881	0.018	50.373	0
Mean slope	Pit volume	0.366	0.067	5.44	5.32×10^{-8}
Mean slope	Rim height	0.646	0.039	16.738	6.96×10^{-63}
Mean slope	Rim radius	-0.153	0.077	-1.978	0.048
Pit depth	RPS	-0.234	0.097	-2.4	0.016
Pit depth	CFT	-0.693	0.038	-18.298	8.58×10^{-75}

(continued)

(continued)

Trait 1	Trait 2	Correlation coefficient (rg)	Standard error	z-score	p-value
Pit depth	Mean slope	0.881	0.018	50.373	0
Pit depth	Pit volume	0.757	0.032	23.428	2.2×10^{-121}
Pit depth	Rim height	0.569	0.044	12.773	2.33×10^{-37}
Pit depth	Rim radius	0.338	0.062	5.447	5.11×10^{-8}
Pit volume	RPS	-0.118	0.085	-1.387	0.166
Pit volume	CFT	-0.786	0.032	-24.557	3.61×10^{-133}
Pit volume	Mean slope	0.366	0.067	5.44	5.32×10^{-8}
Pit volume	Pit depth	0.757	0.032	23.428	2.2×10^{-121}
Pit volume	Rim height	0.137	0.063	2.185	0.029
Pit volume	Rim radius	0.851	0.017	49.399	0
Rim height	RPS	-0.074	0.073	-1.006	0.315
Rim height	CFT	0.198	0.07	2.833	0.005
Rim height	Mean slope	0.646	0.039	16.738	6.96×10^{-63}
Rim height	Pit depth	0.569	0.044	12.773	2.33×10^{-37}
Rim height	Pit volume	0.137	0.063	2.185	0.029
Rim height	Rim radius	-0.115	0.06	-1.94	0.052
Rim radius	RPS	-0.035	0.081	-0.429	0.668
Rim radius	CFT	-0.513	0.049	-10.492	9.36×10^{-26}
Rim radius	Mean slope	-0.153	0.077	-1.978	0.048
Rim radius	Pit depth	0.338	0.062	5.447	5.11×10^{-8}
Rim radius	Pit volume	0.851	0.017	49.399	0
Rim radius	Rim height	-0.115	0.06	-1.94	0.052

A pictorial representation of some of these results can be found in Fig.8.

Supplementary Table S9: Two-sample Mendelian randomization (MR) analyses assessing causal relationships between foveal traits and retinal disease.

Exposure	Outcome	Total number of SNVs	Odds ratio	MRE IVW p-value	weighted median p-value	Egger p-value	weighted mode p-value	Cochran's Q p-value	Egger intercept p-value	Leave-one-out
Rim radius	MTAG AMD	24	1.127	0.777	0.566	0.987	0.678	0.902	0.919	Not consistently significant
Rim radius	POAG	46	0.820	0.551	0.742	0.205	0.612	0.753	0.249	Not consistently significant
Pit volume	POAG	44	0.937	0.016	0.056	0.024	0.034	0.841	0.115	Not consistently significant
Pit volume	MTAG AMD	27	1.120	0.004	0.097	0.139	0.891	0.640	0.554	All significant
MTAG AMD	Rim radius	42	1.000	0.777	0.934	0.922	0.847	0.418	0.724	Not consistently significant
MTAG AMD	Pit volume	38	0.997	0.638	0.331	0.305	0.502	0.374	0.344	Not consistently significant
POAG	Pit volume	37	0.992	0.463	0.659	0.064	0.327	0.646	0.084	Not consistently significant
POAG	Rim radius	38	0.999	0.389	0.474	0.068	0.737	0.569	0.099	Not consistently significant

Each row represents a single Mendelian randomization analysis, with the relevant exposure and outcome specified. The number of SNVs used as instruments for the exposure is indicated. The primary analysis was performed using the multiplicative random-effects inverse variance weighted (MRE IVW) method, with p-values reported alongside those from three robust Mendelian randomization methods: weighted median, MR Egger, and weighted mode. Evidence of heterogeneity (Cochran's Q p-value) and directional pleiotropy (Egger intercept p-value) are also shown. The final column summarizes whether the MRE IVW was consistently significant throughout a leave-one-out analysis.

All instruments were genome-wide significant ($p < 5 \times 10^{-8}$), LD-clumped ($R^2 < 0.001$, 10 Mb window), harmonized between exposure and outcome datasets, and screened for outliers and pleiotropic variants using radial Mendelian randomization and Steiger filtering respectively. Palindromic SNVs with a minor allele frequency (MAF) > 0.42 were excluded to avoid strand ambiguity. Summary statistics for MTAG AMD and POAG were obtained from Han *et al.*[1] and Gharahkhani *et al.*[2], respectively. See Methods for further details.

MTAG, Multi-Trait Analysis of GWAS

Supplementary Table S10. Logistic regression analysis investigating the association between the six studied foveal traits and incident AMD and glaucoma

Foveal trait	Event	Number of cases	Odds ratio	Lower 95% CI	Upper 95% CI	p-value	p-value (FDR)
Pit volume	AMD	1,495	26.118	6.092	111.970	1.12×10^{-5}	6.72×10^{-5}
Rim radius	AMD	1,491	1.859	1.132	3.054	0.014	0.024
Rim height	AMD	1,494	0.995	0.992	0.998	0.002	0.0048
Mean slope	AMD	1,499	0.987	0.952	1.023	0.471	0.5652
Pit depth	AMD	1,499	1.001	0.998	1.003	0.638	0.638
CFT	AMD	1,482	0.996	0.994	0.998	3.02×10^{-5}	1.21×10^{-4}
Pit volume	Glaucoma	1,535	0.191	0.035	1.053	0.057	0.0855
Rim radius	Glaucoma	1,530	0.630	0.378	1.048	0.075	0.100
Rim height	Glaucoma	1,537	0.989	0.986	0.993	9.54×10^{-11}	1.14×10^{-9}
Mean slope	Glaucoma	1,531	0.951	0.918	0.986	0.006	0.012
Pit depth	Glaucoma	1,535	0.996	0.994	0.998	4.96×10^{-5}	1.49×10^{-4}
CFT	Glaucoma	1,532	0.999	0.997	1.002	0.617	0.638

Analyses were performed in a cohort of 55,604 individuals, and all models were adjusted for age at image acquisition, sex, spherical equivalent refractive error and genetic ancestry.

ACKNOWLEDGEMENTS & CONSORTIA

We are very grateful to individuals and families who are included in the UK Biobank for their participation.

We also acknowledge the contribution of the UK Biobank Eye and Vision Consortium. Members of this consortium are: Naomi Allen, Tariq Aslam, Denize Atan, Sarah Barman, Jenny Barrett, Paul Bishop, Graeme Black, Tasanee Braithwaite, Roxana Carare, Usha Chakravarthy, Michelle Chan, Sharon Chua, Alexander Day, Parul Desai, Bal Dhillon, Andrew Dick, Alexander Doney, Cathy Egan, Sarah Ennis, Paul Foster, Marcus Fruttiger, John Gallacher, David Garway-Heath, Jane Gibson, Jeremy Guggenheim, Chris Hammond, Alison Hardcastle, Simon Harding, Ruth Hogg, Pirro Hysi, Pearse Keane, Peng Tee Khaw, Anthony Khawaja, Gerassimos Lascaratos, Thomas Littlejohns, Andrew Lotery, Robert Luben, Phil Luthert, Tom Macgillivray, Sarah Mackie, Savita Madhusudhan, Bernadette Mcguinness, Gareth Mckay, Martin Mckibbin, Tony Moore, James Morgan, Eoin O'Sullivan, Richard Oram, Chris Owen, Praveen Patel, Euan Paterson, Tunde Peto, Axel Petzold, Nikolas Pontikos, Jugnoo Rahi, Alicja Rudnicka, Naveed Sattar, Jay Self, Panagiotis Sergouniotis, Sobha Sivaprasad, David Steel, Irene Stratton, Nicholas Strouthidis, Cathie Sudlow, Zihan Sun, Robyn Tapp, Dhanes Thomas, Emanuele Trucco, Adnan Tufail, Ananth Viswanathan, Veronique Vitart, Mike Weedon, Cathy Williams, Katie Williams, Jayne Woodside, Max Yates, Jennifer Yip, Yalin Zheng.

This research was conducted using the UK Biobank Resource under projects 53144 and 49978.

SUPPLEMENTARY REFERENCES

1. Han, X. *et al.* Genome-wide meta-analysis identifies novel loci associated with age-related macular degeneration. *J Hum Genet* 65, 657–665 (2020).
2. Gharahkhani, P. *et al.* Genome-wide meta-analysis identifies 127 open-angle glaucoma loci with consistent effect across ancestries. *Nat Commun* 12, (2021).
